# Elevated pH Conditions Associated With *Microcystis* spp. Blooms Decrease Viability of the Cultured Diatom *Fragilaria crotonensis* and Natural Diatoms in Lake Erie

**DOI:** 10.3389/fmicb.2021.598736

**Published:** 2021-02-24

**Authors:** Brittany N. Zepernick, Eric R. Gann, Robbie M. Martin, Helena L. Pound, Lauren E. Krausfeldt, Justin D. Chaffin, Steven W. Wilhelm

**Affiliations:** ^1^Department of Microbiology, The University of Tennessee, Knoxville, Knoxville, TN, United States; ^2^F.T. Stone Laboratory and Ohio Sea Grant, The Ohio State University, Put-in-Bay, OH, United States

**Keywords:** microcystis blooms, CyanoHABs, lake alkalinity, biogenic silica, diatoms, Lake Erie

## Abstract

Cyanobacterial Harmful Algal Blooms (CyanoHABs) commonly increase water column pH to alkaline levels ≥9.2, and to as high as 11. This elevated pH has been suggested to confer a competitive advantage to cyanobacteria such as *Microcystis aeruginosa*. Yet, there is limited information regarding the restrictive effects bloom-induced pH levels may impose on this cyanobacterium’s competitors. Due to the pH-dependency of biosilicification processes, diatoms (which seasonally both precede and proceed *Microcystis* blooms in many fresh waters) may be unable to synthesize frustules at these pH levels. We assessed the effects of pH on the ecologically relevant diatom *Fragilaria crotonensis in vitro*, and on a Lake Erie diatom community *in situ*. *In vitro* assays revealed *F. crotonensis* monocultures exhibited lower growth rates and abundances when cultivated at a starting pH of 9.2 in comparison to pH 7.7. The suppressed growth trends in *F. crotonensis* were exacerbated when co-cultured with *M. aeruginosa* at pH conditions and cell densities that simulated a cyanobacteria bloom. Estimates demonstrated a significant decrease in silica (Si) deposition at alkaline pH in both *in vitro F. crotonensis* cultures and *in situ* Lake Erie diatom assemblages, after as little as 48 h of alkaline pH-exposure. These observations indicate elevated pH negatively affected growth rate and diatom silica deposition; in total providing a competitive disadvantage for diatoms. Our observations demonstrate pH likely plays a significant role in bloom succession, creating a potential to prolong summer *Microcystis* blooms and constrain diatom fall resurgence.

## Introduction

Toxin-producing cyanobacteria of the genus *Microcystis* have inundated fresh waters in recent decades (Steffen et al., 2014). Blooms have detrimental ecological and economic effects due to the production of secondary metabolites and the formation of extensive biomass that, upon bloom termination, can drive anoxia (Anderson, 2009). To this end, there is a crucial need to determine the factors responsible for the ecological success of *Microcystis*.

**FIGURE 1:**
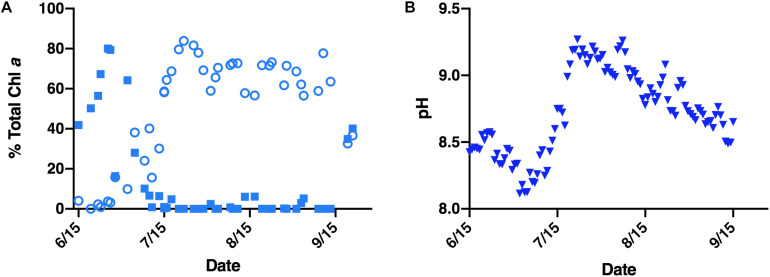
Environmental data corresponding to a 2015 Lake Erie *M. aeruginosa* bloom. **(A)** Relative abundance (reported as percentage of total chlorophyll *a*) of diatoms (closed blue squares) and cyanobacteria (open blue circles) within the Lake Erie water column. **(B)** Average daily pH of the Lake Erie water column (closed blue triangles).

The mechanisms by which *Microcystis* displaces other phytoplankton in fresh waters, including Lake Erie (United States/Canada), Lake Okeechobee (United States) and Lake Tai (China) remain unclear, but are likely multifaceted. In these lakes, a seasonal pattern of phytoplankton taxa succession has emerged. Non-toxic diatoms and other algae dominate throughout fall, winter, and spring, only to be displaced by *Microcystis* blooms mid-summer into fall (Ke et al., 2008; Reavie et al., 2014). This successional trend has been evidenced in Lake Erie’s paleolimnological record, which traces the emergence of eutrophication back to the 1930’s (Allinger and Reavie, 2013). Monitoring efforts of a 2015 *Microcystis* bloom in Lake Erie’s western basin further confirmed this succession, demonstrating diatoms dominated the early summer period prior to their succession by cyanobacteria in mid-summer (Figure 1A).

Several factors contribute to the ecological success of *Microcystis*. Summer dominance in Lake Erie’s western basin has been attributed to nutrient loading (Michalak et al., 2013; Paerl et al., 2016), predation (Vanderploeg et al., 2001; Steffen et al., 2015), and temperature (Andersson et al., 1994; Peng et al., 2018). Likewise, spring diatom decline has been linked to silica limitation and temperature intolerance (Twiss et al., 2012; Reavie et al., 2016). While these factors each contribute to *Microcystis* growth during cyanobacterial bloom years, non-cyanobacterial bloom years have demonstrated that diatoms, such as the temperature tolerant *Fragilaria crotonensis*, can persist and even dominate the summer water column in Lake Erie (Hartig and Wallen, 1986; Saxton et al., 2012; Reavie et al., 2014). Indeed, *F. crotonensis* summer blooms were a frequent occurrence in the western basin of Lake Erie throughout the 1960–1980’s during lake remediation efforts (Hartig, 1987). Furthermore, monitoring data from the 2015 Lake Erie *Microcystis* bloom indicates dissolved silica concentrations, though lowest during the peak diatom bloom, were non-limiting during *Microcystis* succession (Supplementary Figure 1). These observations suggest there are additional and multiple factors contributing to *Microcystis* succession of spring-summer diatoms (Wilhelm et al., 2020). Amongst these factors playing a potential role in succession dynamics is pH. For example, during the 2015 *M. aeruginosa* bloom monitoring efforts, a sharp rise in water column pH was found to co-occur with cyanobacterial bloom formation (Figures 1A,B). While pH can have multiple effects on cellular physiology and biogeochemistry, in the present study, we investigated the response of one physiological aspect of diatoms – silicification – to the shifts in pH that occur during *Microcystis* blooms.

*Microcystis* blooms increase water column pH above 9.2 as CO_2_ is consumed during photosynthesis (Verspagen et al., 2014; Bullerjahn et al., 2016; Krausfeldt et al., 2019). This alkaline pH is considered advantageous to cyanobacteria (Wilson et al., 2010; Shruthi and Rajashekhar, 2014), due to their unique carbon concentrating mechanisms (CCMs) which confer a competitive advantage during growth at low CO_2_ / high pH conditions (Shapiro, 1990; Raven, 2010; Sandrini et al., 2016). Yet freshwater and marine diatoms have been shown to possess a competitive array of CCMs themselves which optimize CO_2_ and HCO_3_^–^ acquisition (Clement et al., 2017). While this may allow diatoms to evade pH-induced carbon-limitation, elevated pH has been shown to decrease carbon uptake, growth rate and metabolic processes in various marine diatoms (Raven, 1981; Taraldsvik and Myklestad, 2000). While these effects of pH on diatom carbon acquisition have been well characterized, pH-induced effects on other metabolic processes have been widely unstudied to date, particularly in freshwater diatoms.

One metabolic process that serves as a distinctive metric for diatom viability is silica deposition. Diatoms possess siliceous cell walls (i.e., frustules) which may pose a unique disadvantage in alkaline bloom conditions. Silica deposition relies on the uptake of dissolved silica (dSi) in the form of silicic acid (Si[OH]_4_) to synthesize biogenic silica (bSi) frustules (Vrieling et al., 1999; Otzen, 2012; Hildebrand et al., 2018). In marine and estuarine systems, diatom viability has been strongly correlated to pH, with studies demonstrating marine diatoms are unable to survive at pH > 8.7 due to silica solubility dynamics and the inhibition of biosilicification (Hansen, 2002; Hervé et al., 2012). Yet, to our knowledge, the effect of pH in freshwater diatom Si deposition remains unassessed.

In this study, we combined lab and field-based experiments to assess the effect of pH on diatom growth rate and silica deposition. As part of this effort, we assessed the effect of pH on *F. crotonensis* growth rate in monoculture and ecologically relevant co-cultures with *M. aeruginosa*. Effects of pH on silica deposition were assessed using a fluorescent dye (PDMPO) which intercalates into newly formed frustules. Laboratory and field-based results indicate pH conditions consistent with *M. aeruginosa* blooms (i.e., pH ≥ 9.2) decreased diatom growth rate, abundance, and silica deposition. In total the pH shift reduces diatom viability and the ability to compete for valuable niche-space with cyanobacteria.

## Materials and Methods

### Assessing Successional Trends of a 2015 Lake Erie *Microcystis* Bloom

A temporal dataset collected at the Ohio State University Stone Laboratory was used to preliminarily evaluate the dynamics of cyanobacteria, diatoms, and pH during the summer of 2015, which was the largest *M. aeruginosa* bloom observed to date (Davis et al., 2019). Water column pH was recorded every 30 min *via* a Yellow Spring Instruments 6600v2 multiprobe sonde suspended at 1 m depth from a buoy located between South Bass and Gibraltar Islands (N 41.66°, W 82.92°). Water samples for phytoplankton community composition were collected next to the buoy several times a week. Diatom-specific and cyanobacteria-specific chlorophyll *a* concentrations were recorded *via* a bbe Moldaenke FluoroProbe (Beutler et al., 2002). Total chlorophyll *a* concentrations corresponding to the sampling period have been provided (Supplementary Figure 2), with the complete details of this dataset found in the original publication (Chaffin et al., 2018).

### Effect of pH on Growth in *F. crotonensis* and *M. aeruginosa* Monocultures

To assess the effects of this environmentally observed pH on diatom and cyanobacteria growth, *in vitro* monoalgal experiments were performed using 2 model taxa. *F. crotonensis* SAG 28.96 (acquired from the *Culture Collection of Algae at the University of Göttingen*, Germany) and *M. aeruginosa* NIES 843 (acquired from the *National Institute for Environmental Studies*, Japan) were maintained in batch cultures using CT medium (Wilhelm, 2017) at respective optimal pH levels of pH 7.7 (Guillard and Lorenzen, 1972; Hervé et al., 2012) and 8.2 (Watanabe et al., 2000; Krausfeldt et al., 2019). To initiate *in vitro* monoculture experiments, *F. crotonensis* and *M. aeruginosa* cultures were filter-concentrated, respectively, using a 2.0-μm and 1.0-μm nominal pore-size 47-mm diameter polycarbonate filter and inoculated into sterile 250 mL filter-vented, baffled polycarbonate flasks (Corning) at a starting concentration of ∼ 700 cells/mL. Monocultures were maintained in 125 mL CT medium containing a non-limiting concentration of silicic acid (176 μM Na_2_SiO_3_ • 9H_2_O) (Hervé et al., 2012) and adjusted to an initial pH of 7.7 (optimal pH for diatom growth) or 9.2 (pH observed during *Microcystis* blooms). pH conditions in the lab study were maintained by adding TAPS buffer as described previously (Zepernick et al., 2020). Cultures were monitored for 30 days at 26°C, with orbital shaking at 70 rpm, and a light intensity of approximately 55–60 μmol photons m^–2^ s^–1^ on a 12:12 light: dark photoperiod cycle.

Abundances were measured every 2-d *via* flow cytometry (BD FACSCalibur). Populations of each species were gated and counted based on forward scatter (FSC), a proxy for size, and chlorophyll *a* fluorescence (FL3) using FlowJo^TM^ software (Becton, Dickinson and Company). Due to the filamentous nature of *F. crotonensis*, direct estimates of individual cell abundance are challenging (Bramburger et al., 2017). In this study, *F. crotonensis* abundances are estimated based on filaments/mL, which form a tight cluster (Supplementary Figure 3). Exponential growth rates (μ)were calculated as the slope of log-scaled data, and were reported in filaments/mL for *F. crotonensis*, and cells/mL for *M. aeruginosa*. Specifically, log-scaled growth data for each replicate were fitted with a linear regression to select time points to be used for μ calculations. Time points demonstrating the logarithmic growth phase with a linear regression R^2^ value of ≥0.95 (i.e., the most linear data points) were subsequently used to calculate average growth rate). Culture pH was checked every 10-d using a sterilized pH probe (Mettler Toledo Seven Compact^TM^ pH/Ion meter S220 fitted with a Mettler InLab Expert Pro-ISM electrode with a temperature range and correction of up to 100°C). Growth experiments were performed in biological triplicate. We note all results will be referred to in this study based on the initial pH condition of the treatment.

### Effect of pH on Growth in *F. crotonensis* and *M. aeruginosa* Co-cultures

To evaluate the effects of ecologically relevant pH conditions on diatom growth, *in vitro* co-culture assays were performed. Concurrent with the monoculture assays, co-cultures of *F. crotonensis* SAG 28.96 and *M. aeruginosa* NIES 843 were inoculated. To initiate *in vitro* co-culture experiments, *M. aeruginosa* and *F. crotonensis* batch cultures were filter-concentrated and inoculated into the same experimental media and initial pH levels as previously described. Taxa were inoculated at 3 ratios (reported as *F. crotonensis:M. aeruginosa)* based on the succession patterns observed (Figure 1): 10:1 ratio simulating a spring diatom bloom, 1:1 ratio simulating the onset of the summer *M. aeruginosa* bloom, and a 1:10 ratio simulating the peak *M. aeruginosa* bloom. All co-cultures were inoculated at net starting concentrations of ∼7,000 cells/mL. Hereafter, co-culture treatments will be referred to by the *F. crotonensis:M. aeruginosa* ratio. Co-cultures were subjected to the same incubation conditions and procedures as described above. All experiments were performed in biological triplicate.

### Effect of pH on *in vitro F. crotonensis* Silica Deposition

To determine the effect of pH on silica deposition *in vitro*, batch cultures of *F. crotonensis* SAG 28.96 were inoculated with the fluorescent dye PDMPO [2-(4-pyridyl)-5-((4-(2-dimethylaminoethylaminocarbamoyl)methoxy)phenyl)oxazole] (Lysosensor DND 160 Yellow/Blue; Invitrogen, Carlsbad, CA, United States). Diatom cultures were acclimated to pH conditions of 7.7 and 9.2 for a 6-d period (i.e., approximately 2 doubling times). Acclimated cultures were filter-concentrated using a 2.0-μm nominal pore-size 47-mm diameter polycarbonate filter and inoculated in acid-clean, sterilized 50 mL glass culture tubes containing 25 mL of CT medium with 176 μ M Na_2_SiO_3_ • 9H_2_O. Tubes were inoculated at an initial concentration of ∼1500 filaments/mL. PDMPO was added at a final concentration of 0.125 μM (Leblanc and Hutchins, 2005). Cultures were incubated at 26°C and approximately 55–60 μmol photons m^–2^ s^–1^ on a 12:12 light: dark photoperiod cycle for 48 h. Abundances were determined *via* flow cytometry as described above.

Si deposition was assessed using microscopic and fluorometric approaches that detect freshly incorporated PDMPO. Bulk Si deposition into individual cells was assessed *via* epifluorescence microscopy. After 48 h, 2 mL of each culture was filtered onto 0.2-μm nominal pore-size 25 mm diameter black polycarbonate filters (Millipore), mounted onto glass slides, treated with anti-fade (Suttle and Fuhrman, 2010), and a coverslip applied prior to storage (−80°C). *F. crotonensis* killed controls (0.5% glutaraldehyde-fixed) were performed according to previous studies (Saxton et al., 2012) to assess abiotic incorporation. Slides were viewed on a Leica DM5500 (Wetzlar, Germany) epifluorescence microscope equipped with a Hamamatsu ORCA-ER camera (Sewickley PA) according to previous methods (Saxton et al., 2012). A “Texas red” filter cube set (λ_ex_ = 520–600 nm; λ_em_ = 570–720 nm) was used to view chlorophyll *a* autofluorescence, and a “DAPI filter” cube set (λ_ex_ = 340–380 nm; λ_em_ > 425 nm) to view PDMPO fluorescence, indicative of Si deposition during the experimental period. Quantitative assessment of epifluorescence microscopy data was achieved by randomized scoring of 100 *F. crotonensis* filaments per pH treatment: analyses included chlorophyll *a* fluorescing cells per filament, PDMPO fluorescing cells per filament, and the proportion of filaments demonstrating ≥one PDMPO fluorescing cells. Total Si deposition was quantitatively measured fluorometrically after HCl-Milli Q lysis to remove unincorporated PDMPO from the silica deposition vesicle (SDV), followed by frustule digestion with hot-NaOH (Saxton et al., 2012; Zepernick et al., 2019). After 48 h of growth, 20 mL of culture was collected on a 0.2-μm nominal pore-size 47-mm diameter polycarbonate filter and subjected to HCl-Milli Q lysis. Filters were flash frozen and stored at −80°C until hot NaOH digestion. After frustule digestion, samples were cooled in an ice bath and neutralized using 1M HCl. PDMPO fluorescence was quantitatively determined using a Turner Designs TD-700 fluorometer fitted with a specialized filter set (λ_ex_ = 360–380 nm: λ_em_ = 522–542 nm, Andover Corporation, Salem, NH, United States). A PDMPO standard curve was generated using PDMPO and NaOH-HCl matrix (Supplementary Figure 4), with the PDMPO concentration converted to Si using a conversion factor of 3230:1 for Si:PDMPO (mol:mol) (Saxton et al., 2012). Total silica deposited into frustules after 48 h (μmol) was normalized to final abundance (filaments/mL). PDMPO experiments were performed with five biological replicates.

### Effect of pH on *in situ* Lake Erie Diatom Community Silica Deposition

To evaluate the effects of pH on silica deposition in natural populations, we queried diatom-enriched communities from Lake Erie with PDMPO under varying pH conditions. Samples were collected in late July of 2019 from the western basin of Lake Erie near the Ohio State University Stone Laboratory on South Bass Island (N 41.69; W 82.79). Water column physiochemistry (temperature = 25.3°C; dissolved oxygen = 7.60 mg/L; pH = 8.63; turbidity = 1.46 NTU; chlorophyll *a* = 0.13 RFU) was recorded prior to sampling using an EXO multiparameter sonde (YSI xylem). Experiments were initiated by enriching for diatoms using a 64-μm mesh phytoplankton net, which was lowered to a depth of ∼7 m. Equal volumes of concentrated seston were diluted with lake water and inoculated into acid washed, rinsed 500 mL polycarbonate bottles. Lake water was buffered using TRIS (4.13 mM final concentration) in accordance with the protocol for freshwater C medium (Watanabe et al., 2000). The experiment consisted of three pH treatments: 7.7, 9.2, and an *in situ* pH control for the sample collection site (pH 8.6). To achieve these pH conditions, samples were incrementally titrated using 1 M HCl or NaOH. PDMPO dye was added at a final concentration of 0.125 μM (Leblanc and Hutchins, 2005), and bottles were placed into an *in situ* mesh incubation chamber for 48 h.

Sample pH and chlorophyll *a* concentration were determined at the initiation (T_0_) and termination (T_f_; 48 h) of the incubation. pH was assessed *via* immediate readings of 15 mL subsamples using a pH probe (Mettler Toledo Seven Compact^TM^ pH/Ion meter S220, fitted with a Mettler InLab Expert Pro-ISM electrode with a temperature range of up to 100°C). Chlorophyll *a* concentration was determined from filtration of 100 mL onto 0.2-μm nominal pore-size 47-mm diameter polycarbonate filters. Samples were extracted in 90% acetone for 24 h at 4°C and assessed on a Turner Designs 10-AU Field Fluorometer (Welschmeyer, 1994). To measure silica deposition, samples were collected by filtering 100 mL of sample onto 0.2-μm nominal pore-size 47-mm diameter polycarbonate filters, followed by the HCl-Milli Q lysis method as described above. Samples were flash frozen in liquid N_2_ and stored at −80°C until further processing. Quantitative Si deposition analyses were performed using hot-NaOH frustule digestion, fluorometry, and subsequent calculations using a fresh standard curve (Supplementary Figure 5; Zepernick et al., 2019). Total silica deposited per bottle after 48 h (μmol) was normalized to chlorophyll *a* concentration (μg/L) (Saxton et al., 2012). Field experiments were performed with four biological replicates.

### Statistical Analyses

Statistical comparisons were made using unpaired two-tailed *t*-tests, ordinary one-way ANOVAs, or ordinary two-way ANOVAs, depending on experimental design. One-way and two-way ANOVA post-hoc multiple comparisons were adjusted using Tukey’s HSD. While *F. crotonenis* and *M. aeruginosa* monoculture and co-culture growth rates are presented separately in this text, all experiments were performed con-currently in the same conditions, and thus have been statistically analyzed using ordinary two-way ANOVAs to compare both pH and abundance (Supplementary Tables 1,2). All analyses were performed using GraphPad’s Prism software (Version 8). For this study, we consider a *p*-value < 0.05 to be significant but have reported all values so the reader may decide (Supplementary Tables 1–4).

## Results

### Role of pH in 2015 Lake Erie Bloom Succession Trends

Monitoring data from a 2015 *M. aeruginosa*-dominated bloom demonstrated that total chlorophyll *a* concentration across the season varied from ∼1–3 μg/L in June to ∼20–120 μg/L in July and August (Chaffin et al., 2018 and Supplementary Figure 2). The pre-cyanobacterial bloom period (June through early July) was dominated by diatoms which form ∼50–80% of the total chlorophyll *a* concentration, whereas cyanobacteria were less than 10% (Figure 1A). The mean daily pH during the corresponding diatom bloom period was between 8.08 and 8.56 (Figure 1B). Conversely, during the cyanobacterial bloom period cyanobacteria dominate, forming ∼56–84% of the chl *a* concentration, whereas diatoms were less than 7% and frequently not detected (Figure 1A). The mean daily pH during the *Microcystis* bloom peaked at ∼9.27 and remained higher than 9 throughout most of August (Figure 1B).

### Alkaline pH Decreases Growth Rate of *F. crotonensis* Monocultures

**FIGURE 2:**
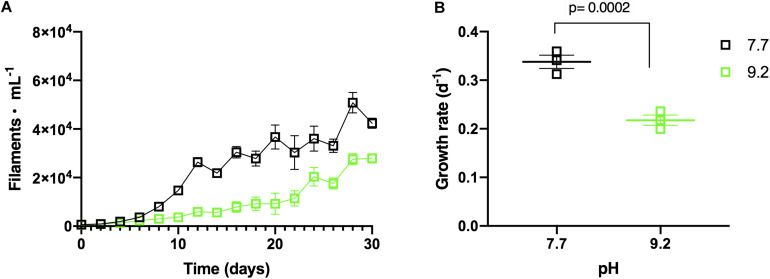
**(A)** *In vitro F. crotonensis* monoculture growth curves at pH 7.7 (black squares) and pH 9.2 (green squares). **(B)** *F. crotonensis* growth rate at pH 7.7 (black squares) and pH 9.2 (green squares). Statistically significant differences between pH treatments are denoted by *p*-values generated by Two-way ANOVAs. Standard error of the mean reported by error bars.

Monoculture experiments demonstrated *F. crotonensis* growth was suppressed at high pH. *F. crotonensis* cultures inoculated at pH 9.2 attained lower abundances throughout the 30-day experiment compared to their pH 7.7 counterparts (Figure 2A). *F. crotonensis* mean growth rate at pH 7.7 was μ = 0.34, with pH 9.2 monocultures exhibiting a significantly lower mean growth rate of μ = 0.22 (*p* = 0.0002) (Figure 2B). Overall, *F. crotonensis* monocultures inoculated at pH 9.2 had a 1.5-fold lower mean growth rate compared to pH 7.7 equivalents.

Effects of pH on *M. aeruginosa* monocultures were less pronounced. *M. aeruginosa* reached higher cell abundances at pH 7.7 compared to pH 9.2 equivalents (Supplementary Figure 6A). Yet, *M. aeruginosa* growth rates were unaffected by pH overall (*p* = 0.503) (Supplementary Figure 6B).

### *M. aeruginosa* Modulates the Effect of pH on *F. crotonensis* in Co-culture

**FIGURE 3:**
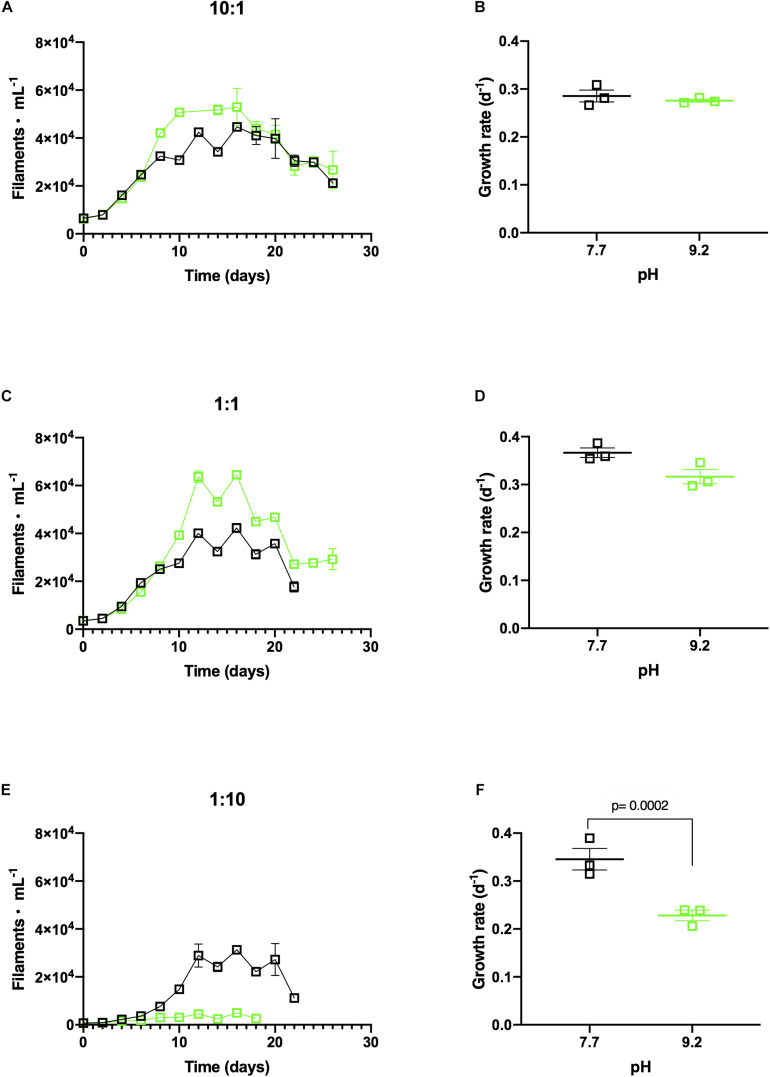
**(A)** *In vitro F. crotonensis* co-culture growth curves in a 10:1 ratio (*F. crotonensis:M. aeruginosa*) at pH 7.7 (black squares) and pH 9.2 (green squares). **(B)** *F. crotonensis* growth rate at 10:1 ratio **(C)** *F. crotonensis* growth curves in a 1:1 ratio **(D)** *F. crotonensis* growth rate in 1:1 ratio **(E)** *F. crotonensis* growth curves in a 1:10 ratio **(F)** *F. crotonensis* growth rate in a 1:10 ratio. Statistically significant differences between pH treatments are denoted by *p*-values generated by Two-way ANOVAs. Standard error of the mean reported by error bars.

*F. crotonensis* reached higher abundances at pH 9.2 than pH 7.7 when co-cultured with non-dominant *M. aeruginosa* concentrations of 10:1 and 1:1 (Figures 3A,C). Additionally, *F. crotonensis* growth rates at the designated pH treatments were not significantly different in the 10:1 ratio (*p* = 0.999) and 1:1 ratio (*p* = 0.206) (Figures 3B,D). Conversely, at the dominant *M. aeruginosa* co-culture ratio of 1:10, *F. crotonensis* growth was substantially suppressed at pH 9.2 (Figure 3E). *F. crotonensis* mean growth rate at pH 7.7 in the 1:10 co-culture was μ = 0.35, with pH 9.2 co-cultures exhibiting a significantly lower mean growth rate of μ=0.23 (*p* = 0.0002) (Figure 3F). Overall, at the 1:10 ratio and pH 9.2, *F. crotonensis* has a 1.5 times lower mean growth rate compared to its pH 7.7 equivalents.

As in the monocultures, the effects of pH on *M. aeruginosa* growth in the co-culture replicates were less pronounced. *M. aeruginosa* reached higher cell concentrations at pH 7.7 in all co-culture ratios compared to pH 9.2 equivalents (Supplementary Figures 7A,C,E). Yet, *M. aeruginosa* growth rates were unaffected by pH overall (*p* > 0.503) (Supplementary Figures 7B,D,F).

### *M. aeruginosa* Concentrations Correspond With pH Increases

While *F. crotonensis* monocultures inoculated at pH 7.7 remained at this pH throughout the 30-day experiment (Supplementary Figure 8), *M. aeruginosa* monocultures demonstrated a steady climb in pH, reaching ∼8.0 by day 30 (Supplementary Figure 9). Similarly, all 3 co-culture ratios inoculated at pH 7.7 demonstrated continual increases in pH throughout the 30-day experiment, reaching final pH levels of ∼8.10 (Supplementary Figure 10). In total, pH 7.7 inoculated *M. aeruginosa* monocultures and co-cultures all experienced increases in pH of ∼0.30, coinciding with increases in *M. aeruginosa* concentrations throughout the 30-day experiment (Supplementary Figures 7,11A). Upon further analysis, *M. aeruginosa* concentrations demonstrated a strong linear relationship with culture pH increases observed in the mono and co-cultures (Simple linear regression R^2^ ≥ 0.8577) (Supplementary Figure 12). Collectively, pH was maintained within a range of approximately +/− 0.40 pH units throughout the 30-day experiment (Supplementary Figure 11).

### Silica Deposition Decreases at Alkaline pH in *F. crotonensis* Monocultures

**FIGURE 4:**
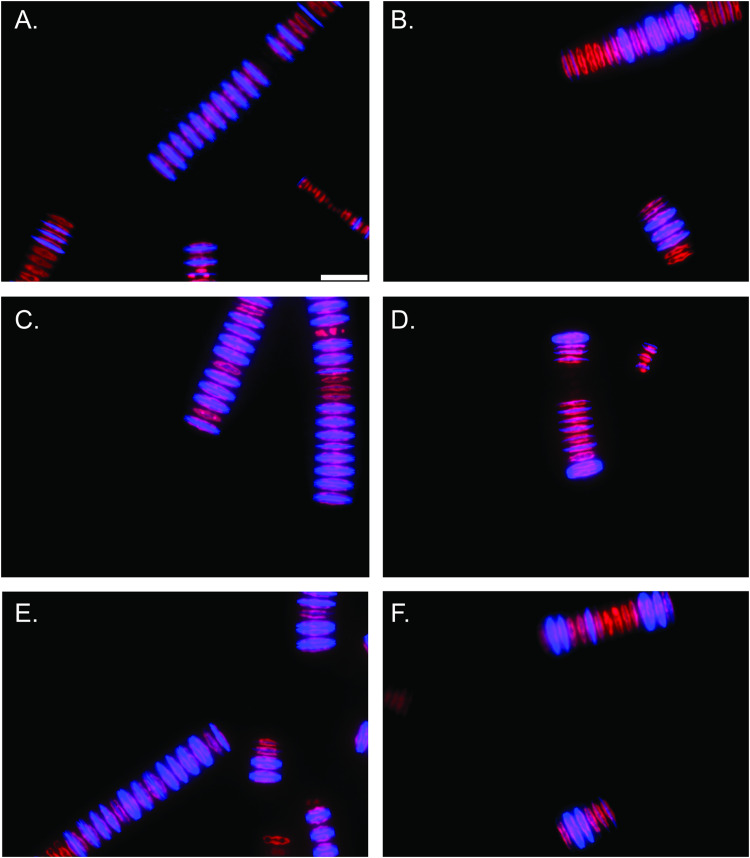
Epifluorescent microscopy images (40x magnification) of *F. crotonensis* filaments after 48 h PDMPO incubations. Scale bar represents 25 μm. Chlorophyll *a* autofluorescence is depicted in red, and PDMPO fluorescence is in blue. **(A,C,E)** *F. crotonensis* cultures acclimated to pH 7.7. **(B,D,F)** *F. crotonensis* cultures acclimated to pH 9.2.

*In vitro* PDMPO incubations demonstrated a pronounced effect of alkaline pH on silica deposition. Epifluorescence microscopy revealed pH 7.7 acclimated cultures deposited more Si after 48 h PDMPO incubations (Figures 4A,C,E and Supplementary Figures 13A,C) compared to pH 9.2 acclimated cultures (Figures 4B,D,F and Supplementary Figures 13B,D). Quantitative counts of these images also demonstrated pH 9.2 acclimated cultures formed significantly smaller filaments than pH 7.7 acclimated cultures (*p* < 0.0001; unpaired two-tailed *t*-test *t* = 4.057, df = 197, *n* = 100) (Supplementary Figure 14A). In total, ∼66% of cells in each filament deposited silica after 48 h in the pH 7.7 treatments, while only ∼30% of the cells in each filament had deposited Si at pH 9.2 (*p* < 0.0001; unpaired two-tailed *t*-test *t* = 9.457, df = 197, *n* = 100) (Supplementary Figure 14B). 100% of *F. crotonensis* filaments incubated at pH 7.7 exhibited at least one diatom cell depositing Si, while only 66% of pH 9.2 *F. crotonensis* filaments demonstrated at least one instance of Si deposition per filament.

**FIGURE 5:**
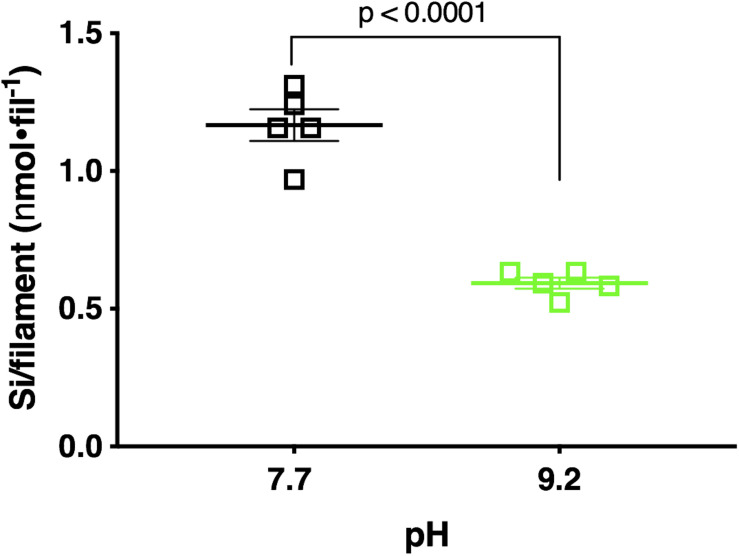
Si deposited per filament after 48 h PDMPO incubations in *F. crotonensis* cultures acclimated to pH 7.7 treatments (black squares) and pH 9.2 (green squares). Statistically significant differences are denoted by respective *p*-values generated by unpaired two-tailed *t*-tests. Standard error of the mean reported by error bars.

Fluorometric data revealed pH 7.7 acclimated cultures deposited a mean of 25.28 μ*mol* Si total, while pH 9.2 acclimated cultures deposited a significantly lower mean of 15.81 μmol Si total (*p* < 0.0001; unpaired two-tailed *t*-test *t* = 8.544, df = 8, *n* = 5) (Supplementary Figure 15). Normalization of this data to abundance (final filament concentration) reflected a similar trend. *F. crotonensis* cultures acclimated to pH 7.7 deposited a mean of 1.17 nmol Si/filament, while cultures acclimated to pH 9.2 deposited a significantly lower mean of 0.59 nmol Si/filament (*p* < 0.0001; unpaired two-tailed *t*-test *t* = 9.446, df = 8, *n* = 5) (Figure 5). Overall, diatoms acclimated to pH 9.2 deposited ∼50% less silica in comparison to their pH 7.7 counterparts.

### Silica Deposition Decreases at Alkaline pH in Lake Erie Diatom Communities

**FIGURE 6:**
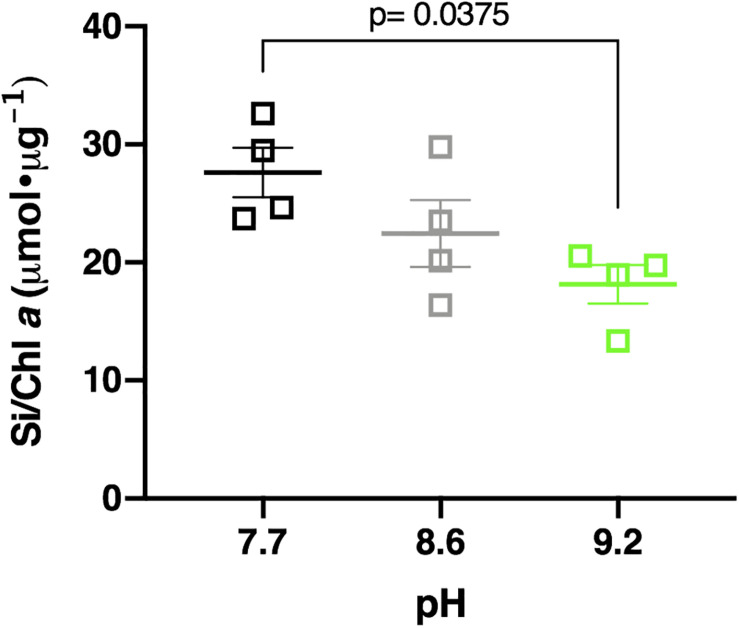
Si deposited per chl *a* concentration in pH 7.7 treatments (black squares), control pH 8.6 (gray squares), and pH 9.2 (green squares) after 48 h incubations. Statistically significant differences are denoted by respective *p*-values generated by One-way ANOVAs. Standard error of the mean reported by error bars.

Elevating the pH negatively influenced Si deposition in the Lake Erie diatom community. Samples incubated at pH 7.7, control pH (8.6), and pH 9.2 deposited a mean of 219.07 μmol Si total, 214.52 μmol Si total, and 194.36 μmol Si total, respectively (Supplementary Figure 16). Total Si deposited in pH 9.2 treatments was less than pH 7.7 treatments, though not statistically significant (*p* = 0.127). Normalization of this data to chlorophyll *a* concentration upheld this observation, with pH 7.7 treatments depositing a mean of 27.61 μmol Si/Chl *a*, control treatments depositing 22.45 μmol Si/Chl *a*, and pH 9.2 treatments depositing 18.16 μmol Si/Chl *a*, respectively (Figure 6). The pH 9.2 treated community deposited significantly less Si per chlorophyll *a* concentration after 48 h compared to the pH 7.7 treated community (*p* = 0.0375). Overall, Lake Erie diatom communities incubated at pH 9.2 deposited ∼1.5 times less Si per chlorophyll *a* concentration than their pH 7.7 counterparts.

## Discussion

Seasonal succession drivers associated with *Microcystis* blooms are complicated. While it remains clear that nutrient-loading results in the planktonic biomass observed during toxic cyanobacterial blooms, the environmental conditions that allow specific organisms to outcompete others are more nuanced (Wilhelm et al., 2020). Here we build on the idea that pH serves as a contributing piece to this puzzle. Previous analyses have suggested a correlation between pH and diatom-*Microcystis* succession in Lake Tai, China (Ke et al., 2008) and Lake Erie (Krausfeldt et al., 2019). In these and other cases, authors have suggested that the effects of pH on carbon acquisition and the superior carbon concentrating mechanisms of cyanobacteria were the major mechanistic drivers of these observations. Additionally, previous studies have indicated nutrient speciation at alkaline pH may favor *Microcystis*, such as the discovery that urea serves as both a carbon and nitrogen source to *M. aeruginosa* at alkaline pH levels (Krausfeldt et al., 2019). While the direct and indirect effects of pH on freshwater diatom carbon and nutrient acquisition cannot be discounted or ruled out, our data demonstrated a previously uncharacterized effect of pH on freshwater diatoms, which may serve to depress them beyond, or in addition to, their ability to acquire CO_2_. We present this information as a factor that likely enhances the exclusion of Si depositing phytoplankton observed during heated summer competition. These observations lead to a take-away message from this study: sometimes it is not the ability of *Microcystis* but the inability of its competitors that results in the taxa succession.

### Effect of pH on Freshwater Diatom Growth

We used pH manipulation in mono- and co-culture experiments to demonstrate that an elevated pH, consistent with *Microcystis*-bloom conditions, negatively affected the diatom *F. crotonensis*. *F. crotonensis* monocultures inoculated at pH 9.2 exhibited lower growth rates and failed to establish a substantial population, demonstrating alkaline pH alone decreases the viability of this model freshwater diatom. Likewise, when co-cultured with dominant concentrations of *M. aeruginosa* at the 1:10 ratio, these alkaline pH effects on abundance were exacerbated. This data is consistent with freshwater diatom decline at the alkaline pH levels observed during summer *Microcystis* blooms. Interestingly, when *F. crotonensis* was co-cultured in the 1:10 ratio at pH 7.7, it was able to maintain growth rates resembling those observed in the pH 7.7 monocultures, suggesting alkaline pH may have a larger role in diatom viability than previously thought. Surprisingly, when *F. crotonensis* was co-cultured with *M. aeruginosa* at 10:1 and 1:1 (i.e., where the diatom biomass dominated) it did not exhibit significant declines in growth rate at pH 9.2. *F. crotonensis* abundances in the 10:1 and 1:1 co-culture were higher at pH 9.2 than their pH 7.7 counterparts, though statistical significance was lacking. While the underlying mechanisms of these results remain unelucidated, this data suggests that while pH is a factor, it alone is likely not the sole driver of diatom exclusion. Another important observation is that in all *M. aeruginosa* mono and co-cultures inoculated at pH 7.7, pH increases in tandem with *M. aeruginosa* cell concentration. This data demonstrates *M. aeruginosa* is indeed capable of driving the pH up substantially despite increased buffer use, and mimics environmental data previously observed during a 2015 *Microcystis* bloom. Cumulatively, *in vitro* co-cultures suggest diatoms may be able to persist in the water column during the spring diatom blooms and onset *Microcystis* blooms regardless of water column pH. Yet, during peak *Microcystis* bloom conditions when the pH is driven to alkaline levels, diatoms are at a disadvantage. This data further suggests these persisting alkaline pH levels may prolong the *Microcystis* bloom period by preventing diatom fall resurgence as a result of decreased diatom growth and viability.

### Effects of pH on Diatom Silica Deposition

Though previous studies have investigated the effects of pH on marine diatom biosilicification (Vrieling et al., 1999; Martin-Jézéquel et al., 2000; Hansen, 2002), this study builds on these observations through an assessment of pH effects on freshwater diatoms. We used PDMPO assays to demonstrate that pH conditions consistent with *Microcystis* blooms significantly decrease silica deposition in both cultured and environmental freshwater diatoms. When interpreting this data it is important to note, flow cytometry analyses of filamentous microorganisms such as *F. crotonensis* count “filaments per volume” rather than “cells per volume.” As a result, an estimate for average number of cells per chain is often used to calculate cells/mL (Bramburger et al., 2017). Our data demonstrate the average number of cells per filament differs significantly in response to culture pH (Supplementary Figure 14), which has the potential to introduce additional error in estimates of cell/mL and biovolume. Indeed, epifluorescence microscopy revealed pH 9.2-acclimated *F. crotonensis* had ∼1.5 times shorter filaments and ∼2 times fewer silica depositing cells per filament. Fluorometric data from the Si deposition assays revealed a similar trend, with *F. crotonensis* cultures acclimated to pH 9.2 depositing ∼50% less silica per filament in comparison to their pH 7.7 counterparts. This trend was further observed in Lake Erie diatom communities, with communities incubated at pH 9.2 depositing ∼1.5 times less Si per chlorophyll *a* concentration than their pH 7.7 counterparts. Cumulatively, this data suggests pH-induced decreases in silica deposition may serve as an important contributor to the freshwater diatom decline observed during *Microcystis* blooms. Furthermore, these results also bring to light a need to further optimize detection and normalization techniques in studies concerning filamentous phytoplankton.

While we have demonstrated a decrease in silica deposition at pH 9.2, the underlying mechanisms remain unclear. Part of our limitation comes from the lack of knowledge concerning functions in the organelle responsible for silica deposition, known as the silica deposition vesicle (SDV). Despite decades of research, the SDV has yet to be isolated or characterized (Martin-Jézéquel et al., 2000; Hildebrand et al., 2018). Additionally, intracellular proteins and pathways associated with diatom biosilicification remain elusive (Thamatrakoln and Hildebrand, 2008; Vardi et al., 2009; Otzen, 2012). External alkaline pH may negatively affect intracellular metabolism in the SDV, which relies on acidic conditions and an undisturbed pH gradient (Vrieling et al., 1999; Hervé et al., 2012). Previous studies have also demonstrated high pH levels may shape intra-cellular diatom silica storage pools (Werner, 1966; Azam et al., 1974; Sullivan, 1977; Martin-Jézéquel et al., 2000). Alkaline pH has previously been shown to limit silica deposition by altering the chemical species of silicic acid or decreasing diatom-uptake rates of dissolved silica (dSi) (Riedel and Nelson, 1985; Amo and Brzezinski, 1999). However, this is unlikely in our study due to the non-limiting concentration of silicic acid in our media (176 μM Na_2_SiO_3_ • 9H_2_O).

In this study, we observed both a decrease in growth rate and silica deposition in response to alkaline pH. Though evidence of a causal link between these two physiological processes is lacking in this study, prior research has established that diatom silica uptake and deposition are tightly coupled with the cell cycle, thus exerting a dependency of silica metabolism on the growth rate (Martin-Jézéquel et al., 2000; Hildebrand et al., 2018). Yet, while silica deposition is essential to diatom viability and their ability to reproduce, diatoms can downregulate silica deposition (i.e., form thinner frustules) to maintain optimal growth rates (Brzezinski et al., 1990; Mcnair et al., 2018). Alternatively, previous research has also demonstrated that as pH increases, growth rates decrease and intracellular silicic acid increases in marine diatoms, potentially indicating a decoupling to silica deposition (Hervé et al., 2012). Hence, pH-associated effects on alternate metabolic processes such as cellular respiration or photosynthesis (i.e., disruptions in normal metabolic regulators) may also contribute to a decline in silica deposition. Furthermore, thinner-frustules have been shown to increase the potential for viral infection and mortality in marine diatoms, exacerbating population declines in the environment (Kranzler et al., 2019). Ultimately, several of these underlying mechanisms may contribute to the decreased silica deposition observed in this study, and further research is needed before any definitive relationship between growth rate, silica deposition, and alkaline pH can be established.

### A Growing Influence of pH in Future Phytoplankton Diversity

Climate change continues to pose a threat to freshwater and marine systems alike. As a result, there is a need to elucidate its effects on factors constraining the ecological success of phytoplankton, such as pH. A recent study has demonstrated ocean acidification has the potential to decrease marine diatom biosilicification rates (Petrou et al., 2019). Conversely, freshwater systems are experiencing a basification attributed to increases in the frequency and duration of HAB events (Wells et al., 2020), which has the potential to decrease freshwater diatom biosilicification. In this manner, the effects of projected pH shifts on phytoplankton succession serve as a critical point of study for ensuring the integrity of global aquatic systems (Flynn et al., 2015; Wells et al., 2020). Our results build on these previous studies, demonstrating pH may play a pivotal role not only in cyanobacterium-driven diatom decline, but phytoplankton taxa diversity in general. While previous studies have demonstrated alkaline bloom-induced pH can serve as a positive feedback mechanism for *M. aeruginosa* (Krausfeldt et al., 2019), this work builds on these efforts by demonstrating these same conditions can facilitate the exclusion of siliceous algae (diatoms). Furthermore, while Lake Erie summer cyanobacterial blooms drive up the western basin pH to an average of ≥9.2, previous winter surveys demonstrate the diatom-dominated water column remains at an average of ∼7.8–8.2 despite fluctuations in chlorophyll *a* and sampling location (Supplementary Figure 17), though additional surveys are needed concerning winter diatom blooms. Cumulatively, this data suggests a role of pH on both the inter and intra-season shifts of phytoplankton taxa within Lake Erie and demonstrate the need to further assess the role of pH in phytoplankton succession.

We noted our pH co-cultures of 10:1 and 1:1 yield higher peak diatom abundance at pH 9.2 in comparison to pH 7.7, despite the diatom monoculture yielding markedly lower abundances at the same elevated pH. In this manner, there may be a window of opportunity for diatoms to persist, and even benefit at low densities of *M. aeruginosa* if the cyanobacterial populations do not become dominant. Many other biological / biogeochemical processes (e.g., inorganic carbon cycling, nitrogen speciation, trace metal chemistry) are pH sensitive and likely play a role in shaping the outcomes of competition for niche space between phototrophs in fresh waters. Our observations serve as a salient reminder that competition in aquatic systems is condition dependent and often complicated by a mix of factors (Wilhelm et al., 2020).

In this study, we confirmed that pH levels of 9.2 decreased diatom growth rate in the filamentous diatom *F. crotonensis.* Our data further demonstrated that silica deposition in lab cultures and environmental diatom communities declined at alkaline pH levels. Cumulatively, these effects reduce diatom viability and fitness in the competition against *Microcystis* blooms. While the pH shift itself may not be sufficient to exclude the diatoms from this (or any) system, the resulting decrease in competitive ability for carbon and nutrients, in addition to pressure from other factors including top-down regulators such as predators and viruses (Kranzler et al., 2019; Pound et al., 2020) appear to tip the scale to favor the cyanobacteria. What remains to be determined beyond this study is how these pressures allow a specific genus of cyanobacteria to proliferate while in competition with many others.

## Data Availability Statement

The original contributions presented in the study are included in the article/Supplementary Material, further inquiries can be directed to the corresponding author/s.

## Author Contributions

BZ and SW designed the experiments. BZ and LK performed preliminary culture optimizations and experimental planning. BZ and EG conducted *in vitro* co-culture assays and performed epifluorescence microscopy. BZ conducted *in vitro* silica deposition assays and performed statistical analyses. BZ, HP, and RM conducted *in situ* Lake Erie silica deposition assays with logistical support from JC. JC performed data collection and analyses corresponding to the 2015 *M. aeruginosa* bloom in Figure 1. All authors contributed to the drafting of the manuscript.

## Conflict of Interest

The authors declare that the research was conducted in the absence of any commercial or financial relationships that could be construed as a potential conflict of interest.
