# Supplementary material for: Elevated pH Conditions Associated With Microcystis spp. Blooms Decrease Viability of the Cultured Diatom Fragilaria crotonensis and Natural Diatoms in Lake Erie
Source: Front Microbiol. 2021 Feb 24;12:598736. doi: 10.3389/fmicb.2021.598736 (PMC7943883; doi:10.3389/fmicb.2021.598736)
Supplement: Supplementary file 1 [file Data_Sheet_1.pdf]

SUPPLEMENTAL ONLINE INFORMATION

For publication in conjunction with the following:

Elevated pH conditions associated with *Microcystis* spp. blooms decrease viability of the cultured diatom *Fragilaria crotonensis* and natural diatoms in Lake Erie

Brittany N. Zepernick[1], Eric R. Gann[1], Robbie M. Martin[1], Helena L. Pound[1], Lauren E. Krausfeldt[1†], Justin D. Chaffin[2], Steven W. Wilhelm[1*]

[1]Department of Microbiology, University of Tennessee Knoxville, 37996, USA
[2]F.T. Stone Laboratory and Ohio Sea Grant, The Ohio State University, Put-In-Bay, OH, United States
[†] current address: Department of Biological Sciences, Halmos College of Arts and Sciences, Nova Southeastern University, Dania Beach, FL 33004, USA
*** Correspondence:**
Steven W. Wilhelm
wilhelm@utk.edu

Supplemental Table 1: Statistical analysis of *F. crotonensis* growth rate in pH mono and co-culture assays. Statistical analyses performed using ordinary two-way ANOVA with Tukey's HSD multiple comparisons test.

Treatment comparison	Summary	P value
pH 7.7 10:1 vs. pH 7.7 1:1	**	0.0098
pH 7.7 10:1 vs. pH 7.7 1:10	ns	0.0815
pH 7.7 10:1 vs. pH 7.7. mc	ns	0.1696
pH 7.7 10:1 vs. pH 9.2 10:1	ns	0.9994
pH 7.7 10:1 vs. pH 9.2 1:1	ns	0.7142
pH 7.7 10:1 vs. pH 9.2 1:10	ns	0.1053
pH 7.7 10:1 vs. pH 9.2 mc	*	0.0378
pH 7.7 1:1 vs. pH 7.7 1:10	ns	0.9450
pH 7.7 1:1 vs. pH 7.7 mc	ns	0.7800
pH 7.7 1:1 vs. pH 9.2 10:1	**	0.0037
pH 7.7 vs. 1:1 vs. pH 9.2 1:1	ns	0.2063
pH 7.7 1:1 vs. pH 9.2 1:10	****	<0.0001
pH 7.7 1:1 vs. pH 9.2 mc	****	<0.0001
pH 7.7 1:10 vs. pH 7.7 mc	ns	0.9998
pH 7.7 1:10 vs. pH 9.2 10:1	*	0.0316
pH 7.7 1:10 vs. pH 9.2 1:1	ns	0.7745
pH 7.7 1:10 vs. pH 9.2 1:10	***	0.0002
pH 7.7 1:10 vs. pH 9.2 mc	****	<0.0001
pH 7.7 mc vs. pH 9.2 10:1	ns	0.0698
pH 7.7 mc vs. pH 9.2 1:1	ns	0.9423
pH 7.7 mc vs. pH 9.2 1:10	***	0.0005
pH 7.7 mc vs. pH 9.2 mc	***	0.0002
pH 9.2 10:1 vs. pH 9.2 1:1	ns	0.4212
pH 9.2 10:1 vs. pH 9.2 1:10	ns	0.2443
pH 9.2 10:1 vs. pH 9.2 mc	ns	0.0965
pH 9.2 1:1 vs. pH 9.2 1:10	**	0.0045
pH 9.2 1:1 vs. pH 9.2 mc	**	0.0015
pH 9.2 1:10 vs. pH 9.2 mc	ns	0.9990

ANOVA Table	SS	DF	MS	F (DFn, DFd)	P value
Interaction	0.01314	3	0.004379	F (3,16) = 8.2	0.0015
pH	0.03311	1	0.03311	F (1,16) = 62	<0.0001
Ratio	0.01637	3	0.005456	F (3,16) = 10	0.0005
Residual	0.008490	16	0.0005306		

Supplemental Table 2: Statistical analysis of *M. aeruginosa* growth rate in pH mono and co-culture assays. Statistical analyses performed using ordinary two-way ANOVA with Tukey's HSD multiple comparisons test.

**Treatment comparison**	**Summary**	**P value**
pH 7.7 10:1 vs. pH 7.7 1:1	ns	0.8850
pH 7.7 10:1 vs. pH 7.7 1:10	ns	0.3390
pH 7.7 10:1 vs. pH 7.7 mc	*	0.0175
pH 7.7 10:1 vs. pH 9.2 10:1	ns	0.5031
pH 7.7 10:1 vs. pH 9.2 1:1	ns	0.1299
pH 7.7 10:1 vs. pH 9.2 1:10	ns	0.1243
pH 7.7 10:1 vs. pH 9.2 mc	**	0.0018
pH 7.7 1:1 vs. pH 7.7 1:10	ns	0.9643
pH 7.7 1:1 vs. pH 7.7 mc	ns	0.1893
pH 7.7 1:1 vs. pH 9.2 10:1	ns	0.9954
pH 7.7 1:1 vs. pH 9.2 1:1	ns	0.7273
pH 7.7 1:1 vs. pH 9.2 1:10	ns	0.7129
pH 7.7 1:1 vs. pH 9.2 mc	*	0.0231
pH 7.7 1:10 vs. pH 7.7 mc	ns	0.6945
pH 7.7 1:10 vs. pH 9.2 10:1	ns	>0.9999
pH 7.7 1:10 vs. pH 9.2 1:1	ns	0.9982
pH 7.7 1:10 vs. pH 9.2 1:10	ns	0.9977
pH 7.7 1:10 vs. pH 9.2 mc	ns	0.1504
pH 7.7 mc vs. pH 9.2 10:1	ns	0.5118
pH 7.7 mc vs. pH 9.2 1:1	ns	0.9527
pH 7.7 mc vs. pH 9.2 1:10	ns	0.9581
pH 7.7 mc vs. pH 9.2 mc	ns	0.9325
pH 9.2 10:1 vs. pH 9.2 1:1	ns	0.9794
pH 9.2 10:1 vs. pH 9.2 1:10	ns	0.9761
pH 9.2 10:1 vs. pH 9.2 mc	ns	0.0876
pH 9.2 1:1 vs. pH 9.2 1:10	ns	>0.9999
pH 9.2 1:1 vs. pH 9.2 mc	ns	0.3813
pH 9.2 1:10 vs. pH 9.2 mc	ns	0.3943

**ANOVA Table**	**SS**	**DF**	**MS**	**F (DFn, DFd)**	**P value**
Interaction	0.0006180	3	0.0002060	F (3,16) = 0.3550	0.7862
pH	0.004290	1	0.004290	F (1,16) = 7.393	0.0152
Ratio	0.01563	3	0.005212	F (3,16) = 8.981	0.0010
Residual	0.009285	16	0.0005803		

Supplemental Table 3: Statistical analysis of *in vitro F. crotonensis* Si deposition assay. **(A)** Comparison of *F. crotonensis* cell concentration as a function of pH. Statistical analyses performed using unpaired two-tailed t-test (t=6.705, df=8, n=5). **(B)** Comparison of total Si deposited as a function of pH. Statistical analyses performed using unpaired two-tailed t-test (t=8.544, df=8, n=5). **(C)** Comparison of Si deposited per filament as a function of pH. Statistical analyses performed using unpaired two-tailed t-test (t=9.446, df=8, n=5). **(D)** Comparison of epifluorescence microscopy *F. crotonensis* cell number per filament and % of cells fluorescing PDMPO per filament as a function of pH. Statistical analyses performed using unpaired two-tailed t-test: #cells/filament: (t=4.057, df=197, n=100), %PDMPO cells/filament: (t=9.457, df=197, n=100).

**A.) Treatment comparison**	**Summary**	**P value**
pH 7.7 filaments/mL vs. pH 9.2 filaments/mL	***	p=0.0002

**B.) Treatment comparison**	**Summary**	**P value**
pH 7.7 total Si vs. pH 9.2 total Si	****	p<0.0001

**C.) Treatment comparison**	**Summary**	**P value**
pH 7.7 Si/filament vs. pH 9.2 Si/filament	****	p<0.0001

**D.) Treatment comparison**	**Summary**	**P value**
pH 7.7 vs. pH 9.2 # cells/filament	****	p<0.0001
pH 7.7 vs. pH 9.2 % PDMPO cells/filament	****	p<0.0001

Supplemental Table 4: Statistical analysis of *in situ* Lake Erie Si deposition assay**. (A)** Comparison of community chlorophyll *a* concentration as a function of pH. Statistical analyses performed using ordinary one-way ANOVA with Tukey's multiple comparisons test. **(B)** Comparison of total Si deposited as a function of pH. Statistical analyses performed using ordinary one-way ANOVA with Tukey's multiple comparisons test. **(C)** Comparison of Si deposited per chlorophyll *a* concentration as a function of pH. Statistical analyses performed using ordinary one-way ANOVA with Tukey's multiple comparisons test.

**A.) Treatment comparison**	**Summary**	**P value**
control 48 hr vs. pH 7.7 48 hr chlorophyll *a*	ns	0.7187
pH 7.7 48 hr vs. pH 9.2 48hr chlorophyll *a*	ns	0.4464
pH 9.2 48 hr vs. control 48hr chlorophyll *a*	ns	0.9614

**B.) Treatment comparison**	**Summary**	**P value**
Lake Erie diatom control vs. pH 7.7 Net Si	ns	0.916
Lake Erie diatom pH 7.7 vs. pH 9.2 Net Si	ns	0.127
Lake Erie diatom pH 9.2 vs. control Net Si	ns	0.229

**C.) Treatment comparison**	**Summary**	**P value**
Lake Erie diatom control vs. pH 7.7 Si/chl *a*	ns	0.285
Lake Erie diatom pH 7.7 vs. pH 9.2 Si/chl *a*	*	0.037
Lake Erie diatom 9.2 vs. control Si/chl *a*	ns	0.404

A.)

**ANOVA Table**	**SS**	**DF**	**MS**	**F (DFn, DFd)**	**P value**
Treatment (between columns)	18.84	2	9.422	F(2,9)=0.2205	0.2205
Residual (within columns)	47.19	9	5.244		
Total	66.04	11			

B.)

**ANOVA Table**	**SS**	**DF**	**MS**	**F (DFn, DFd)**	**P value**
Treatment (between columns)	1383	2	691.7	F(2,9)=2.706	0.1202
Residual (within columns)	2301	9	255.7		
Total	3684	11			

C.)

**ANOVA Table**	**SS**	**DF**	**MS**	**F (DFn, DFd)**	**P value**
Treatment (between columns)	179.3	2	89.63	F(2,9)=4.444	0.0454
Residual (within columns)	181.5	9	20.17		
Total	360.8	11			

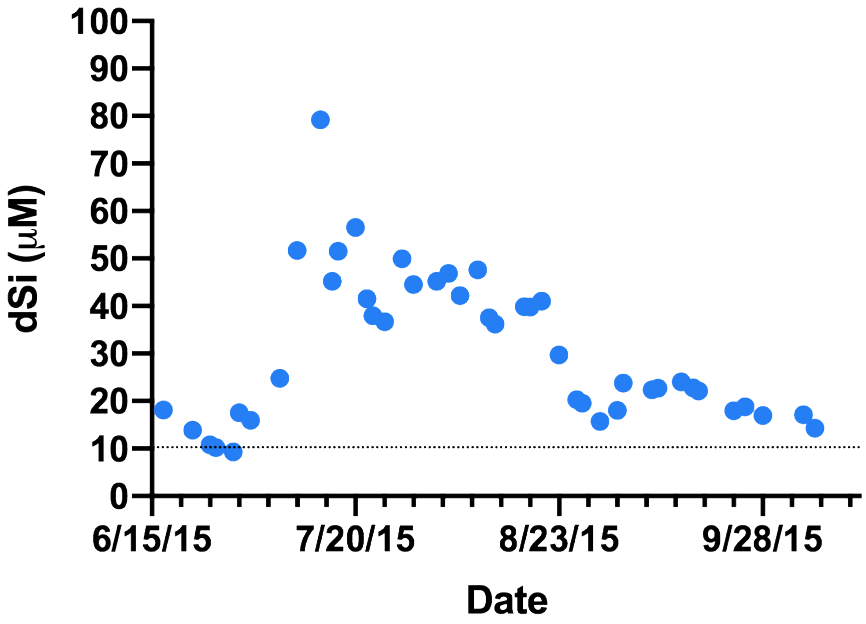

Supplemental Figure 1: Dissolved silica profiles corresponding to a 2015 Lake Erie *M. aeruginosa* bloom in the western basin. Silica-limiting concentrations (defined in this study as 10μM) are indicated by the dotted black line. Complete details of this dataset can be found in (Chaffin et al., 2018).

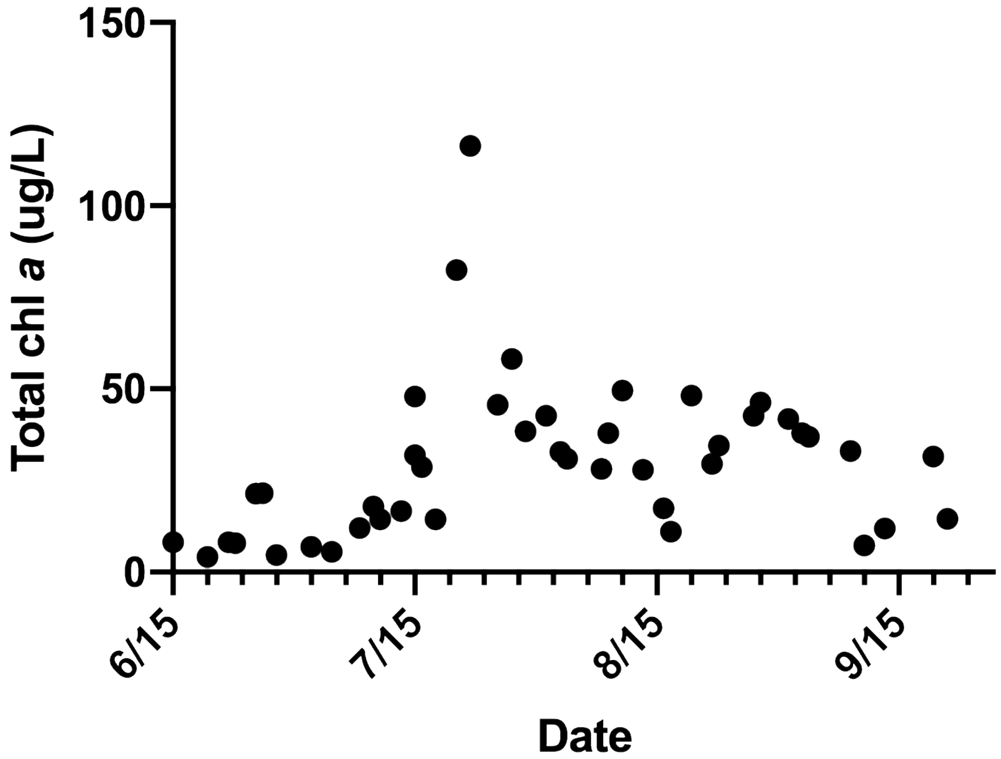

Supplemental Figure 2: Total chlorophyll *a* concentration corresponding to a 2015 Lake Erie *M. aeruginosa* bloom in the western basin. Complete details of this dataset can be found in (Chaffin et al., 2018).

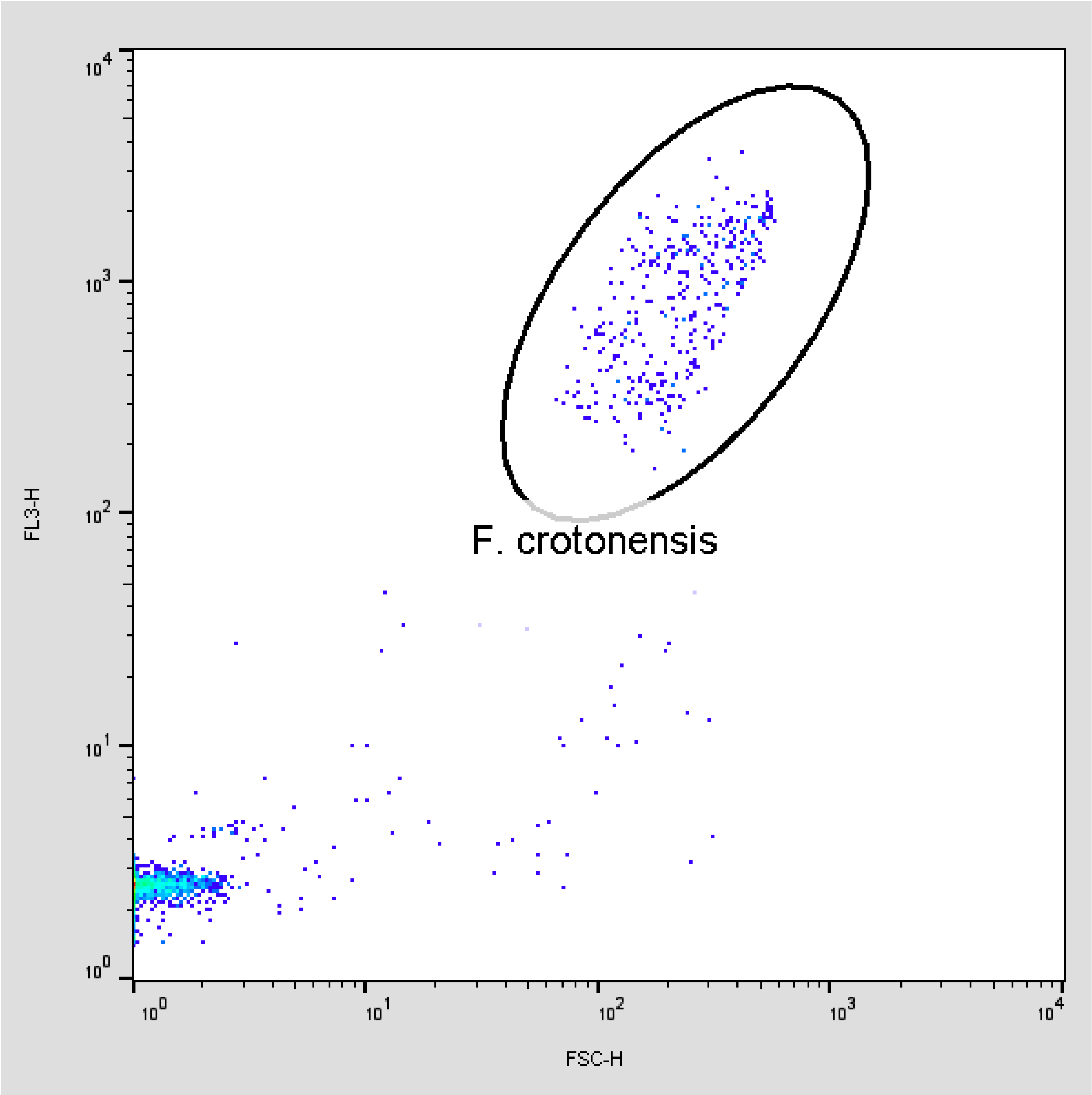

Supplemental Figure 3: FlowJo graph depicting a gated population of *F. crotonensis* filaments run on the flow cytometer. Sample analyzed was a pH 7.7 *F. crotonensis* monoculture culture from 20-d. Background noise visible in lower graph quadrant results from CT media.

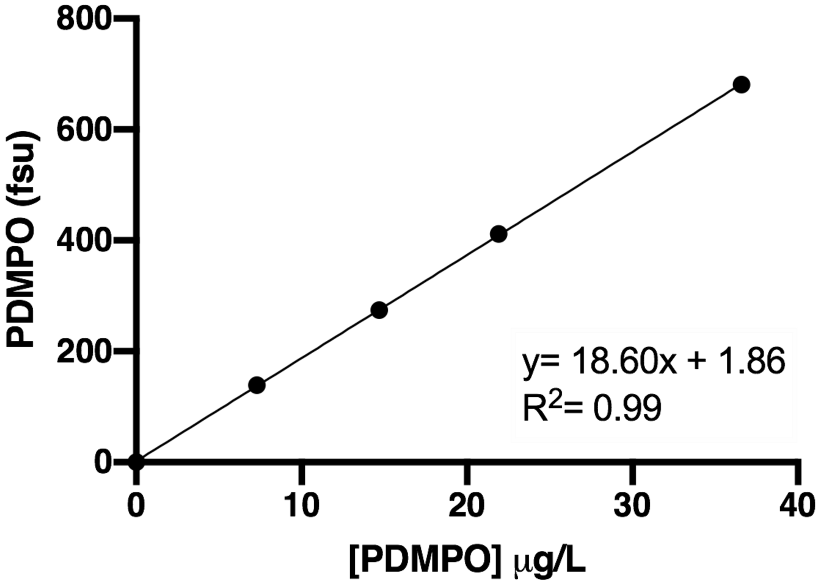

Supplemental Figure 4: (A) Standard curve for *in vitro F. crotonensis* Si deposition assay used to obtain silica deposited per cell reported in Figure 5. Simple linear regression (R^2=0.9999, F=53417, p<0.0001, n=5).

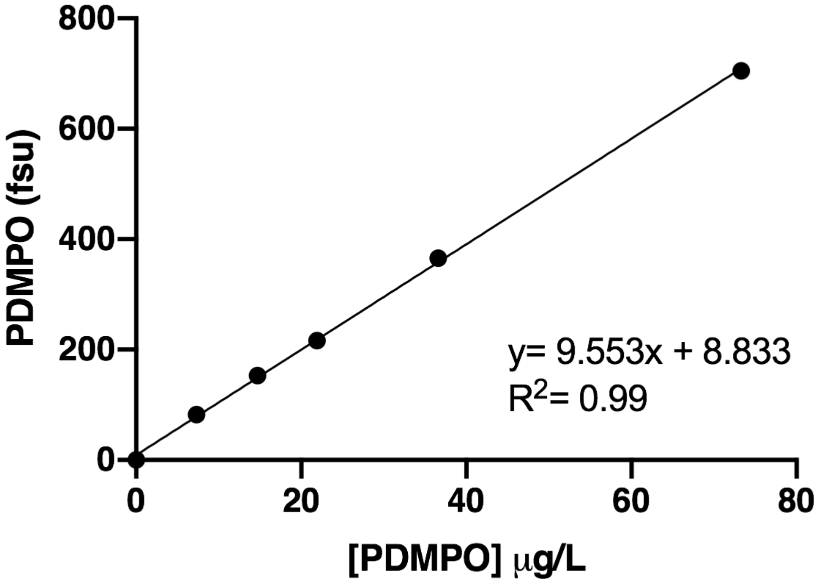

Supplemental Figure 5: Standard curve for *in situ* Lake Erie Si deposition assay used to obtain silica deposited per chlorophyll *a* concentration reported in Figure 6. Simple linear regression (R^2=0.9995, F=7368, p<0.0001, n=6).

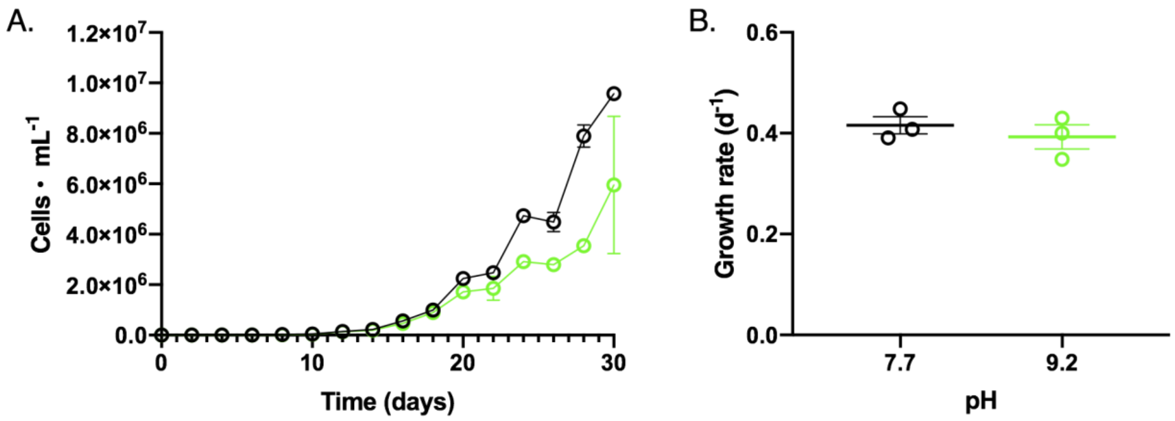

Supplemental Figure 6: (A) *In vitro M. aeruginosa* monoculture growth curves at pH 7.7 (black circles) and pH 9.2 (green circles). **(B)** *M. aeruginosa* growth rate at pH 7.7 (black circles) and pH 9.2 (green circles). Statistically significant differences between pH treatments are denoted by p values generated by Two-way ANOVAs. Standard error of the mean reported by error bars.

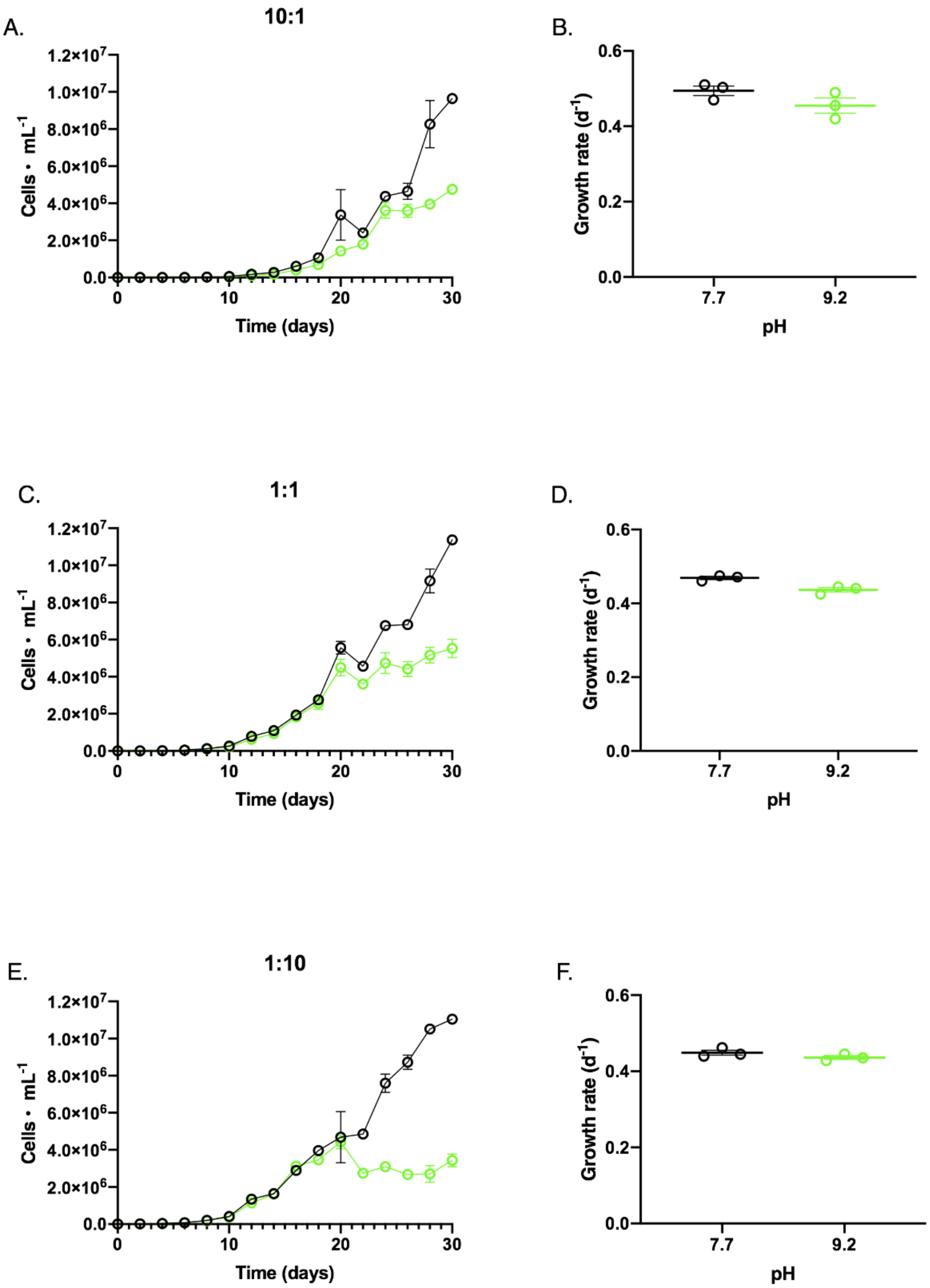

Supplemental Figure 7: (A) *In vitro M. aeruginosa* growth curves in a 10:1 ratio *(F. crotonensis:M. aeruginosa)* at pH 7.7 (black circles) and pH 9.2 (green circles). **(B)** *M. aeruginosa* growth rate in a 10:1 ratio **(C)** *M. aeruginosa* growth curves in a 1:1 ratio **(D)** *M. aeruginosa* growth rate in a 1:1 ratio **(E)** *M. aeruginosa* growth curves in a 1:10 ratio **(F)** *M. aeruginosa* growth rate in a 1:10 ratio. Statistically significant differences between pH treatments are denoted by p values generated by Two-way ANOVAs. Standard error of the mean reported by error bars.

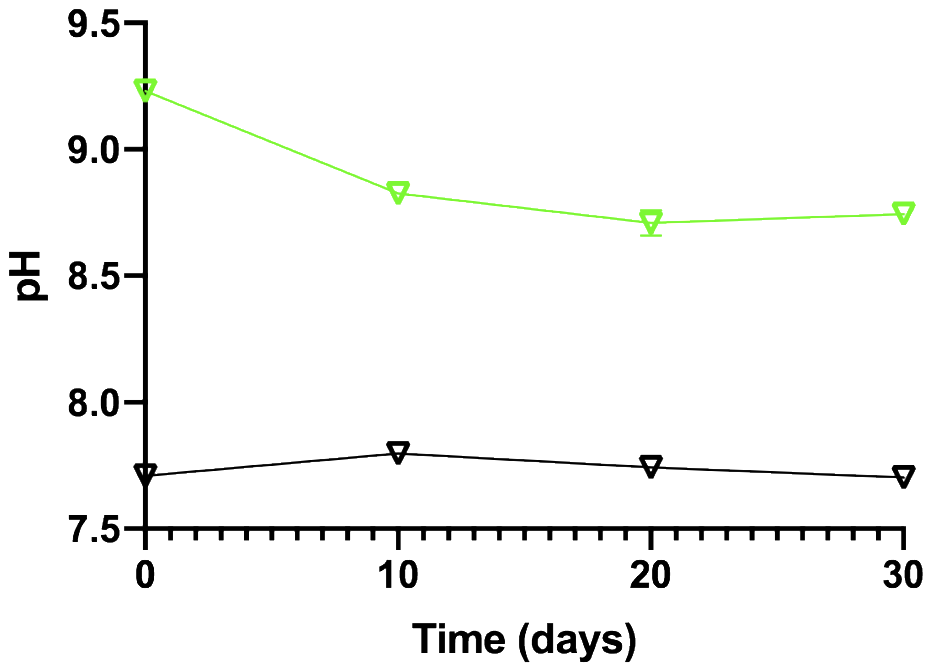

Supplemental Figure 8: pH drift in F. *crotonensis* monocultures inoculated at pH 7.7 (black inverted triangles) and pH 9.2 (green inverted triangles). Standard error of the mean reported by error bars.

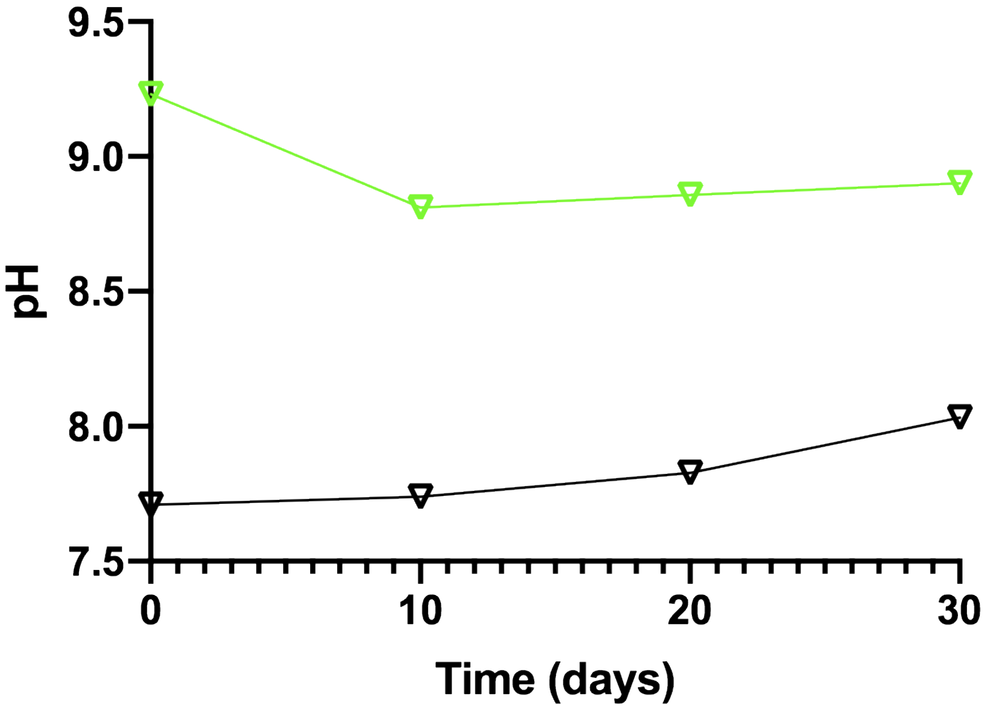

Supplemental Figure 9: pH drift from initial pH in *M. aeruginosa* monocultures inoculated at pH 7.7 (black inverted triangles) and pH 9.2 (green inverted triangles). Standard error of the mean reported by error bars.

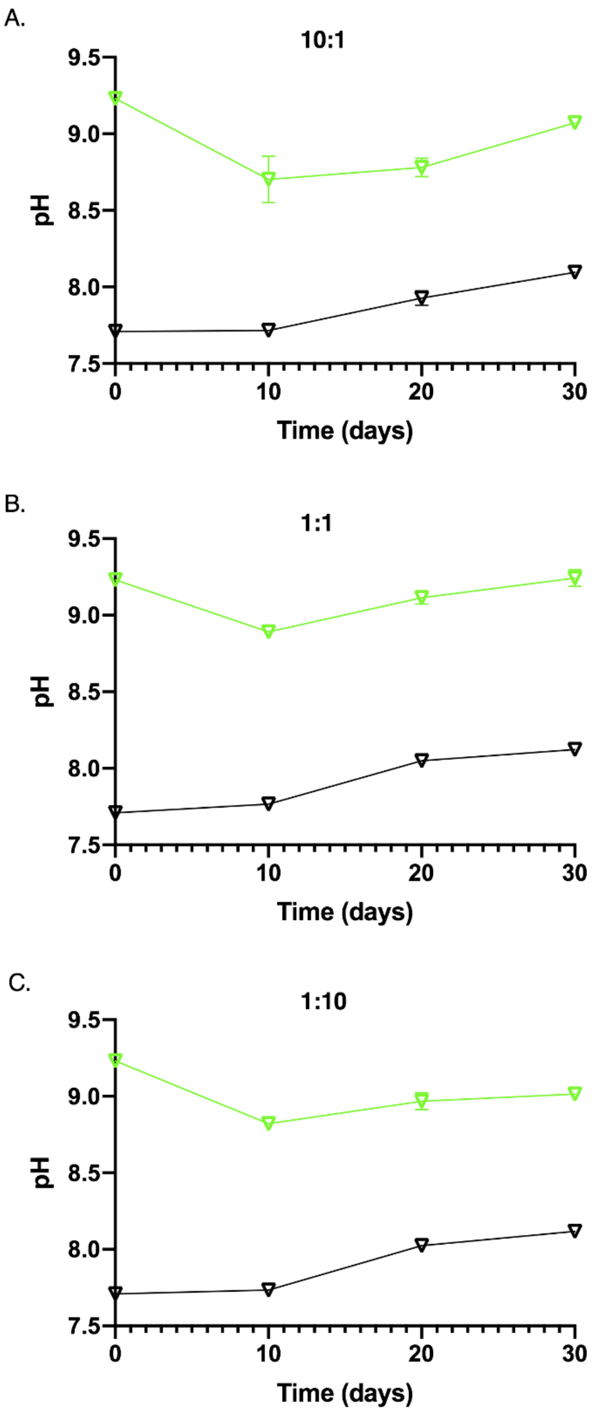

Supplemental Figure 10: pH drift from initial pH in co-cultures inoculated at pH 7.7 (black inverted triangles) and pH 9.2 (green inverted triangles). Standard error of the mean reported by error bars.

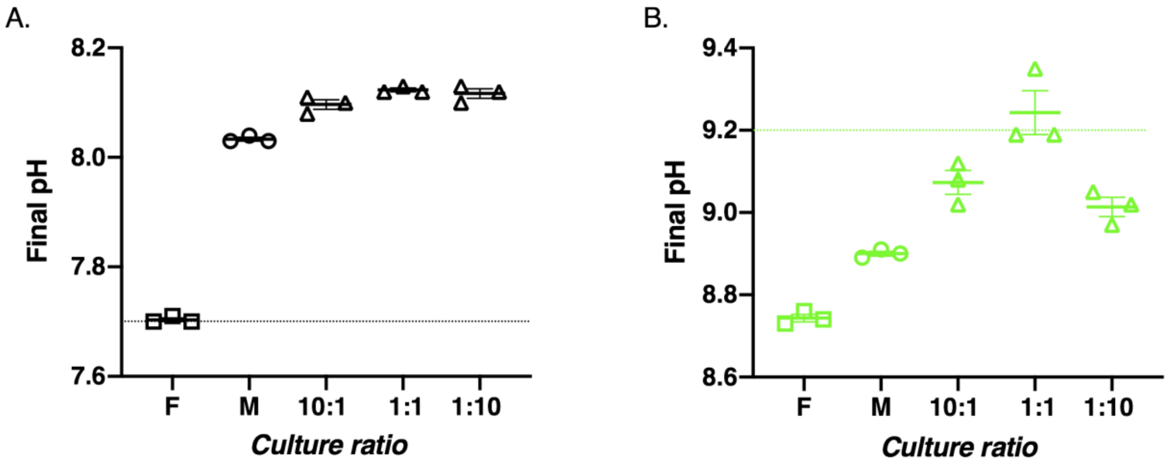

Supplemental Figure 11: Final pH of all mono and co-cultures after 30-d. **(A)** Final pH of pH 7.7. treatment cultures (black) of *F. crotonensis* monocultures (squares), *M. aeruginosa* monocultures (circles) and co-cultures (triangles). **(B)** Final pH of pH 9.2 treatment cultures (green) of *F. crotonensis* monocultures (squares), *M. aeruginosa* monocultures (circles) and co-cultures (triangles). Initial pH levels at 0-d are indicated by dotted lines. Standard error of the mean reported by error bars.

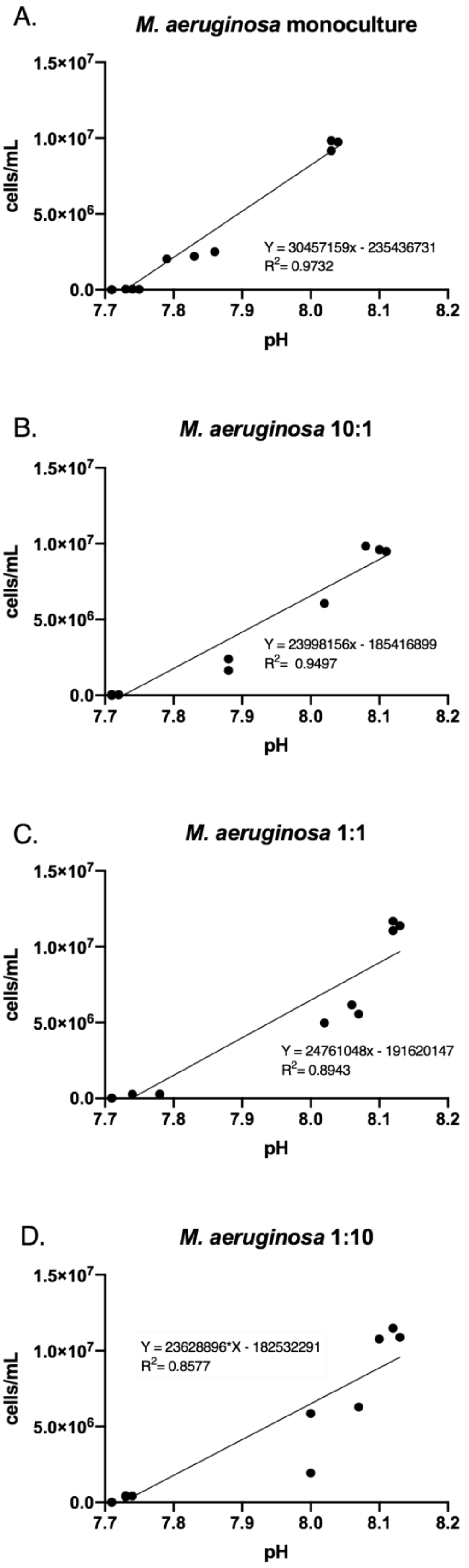

Supplemental Figure 12: Simple linear regressions of *M. aeruginosa* concentration and pH drift of mono **(A)** and co-cultures **(B, C, D)** inoculated at pH 7.7. Cell concentrations and pH levels were sampled at 0, 10, 20, and 30 d. **(A)** Simple linear regression: R^2=0.9732, F=363.4,p<0.0001, n=12, **(B)** Simple linear regression: R^2=0.9497, F=188.9,p<0.0001,n=12, **(C)** Simple linear regression: R^2=0.8943, F=84.60, p<0.0001, n=12, **(D)** Simple linear regression: R^2=0.8577, F=60.29, p<0.0001, n=12).

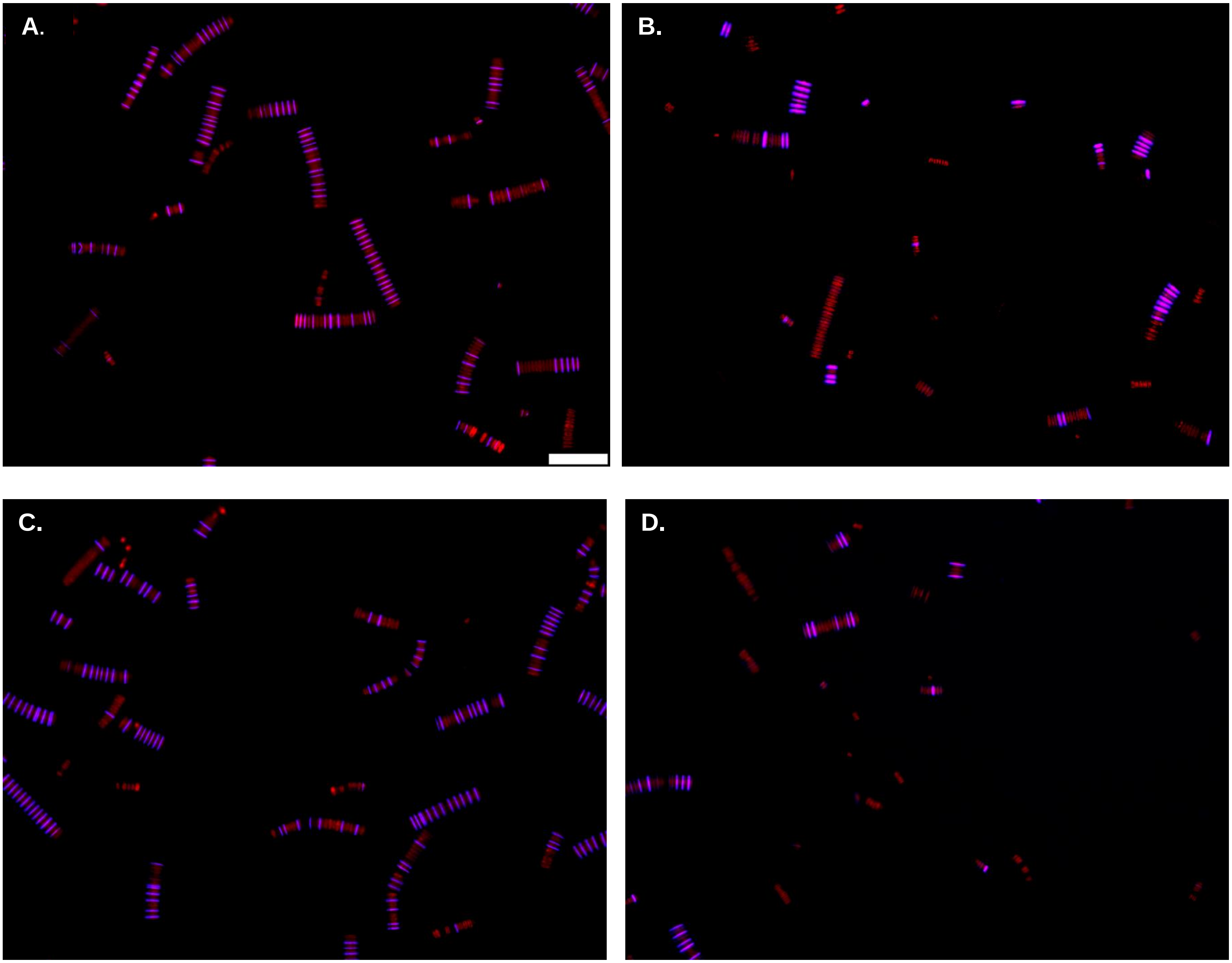

Supplemental Figure 13: Epifluorescent microscopy images (10x magnification) of *in vitro F. crotonensis* filaments after 48 h PDMPO incubations. Scale bar represents 75 μm (depicted in panel A). Chlorophyll *a* autofluorescence is depicted in red, and PDMPO fluorescence is indicated in blue. **(A, C)** *F. crotonensis* chains acclimated to pH 7.7. **(B, D)** *F. crotonensis* filaments acclimated pH 9. 2.

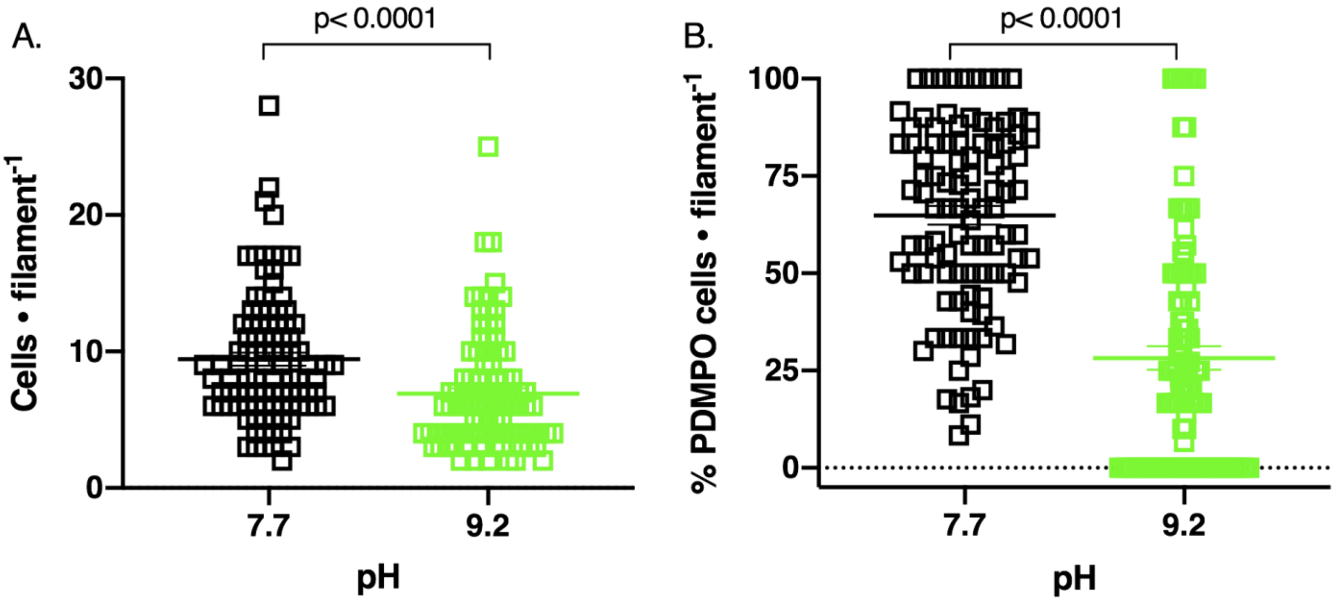

Supplemental Figure 14: Epifluorescent microscopy data of *F. crotonensis* Si deposition assay after 48 h PDMPO incubations. **(A)** Number of cells per filament in pH 7.7 acclimated (black squares) and pH 9.2 acclimated (green squares) *F. crotonensis* cultures. **(B)** Percentage of cells fluorescing PDMPO per filament in pH 7.7 acclimated (black squares) and pH 9.2 acclimated (green squares) *F. crotonensis* cultures. Statistically significant differences are denoted by respective p values generated by unpaired two-tailed t-tests. Standard error of the mean reported by error bars.

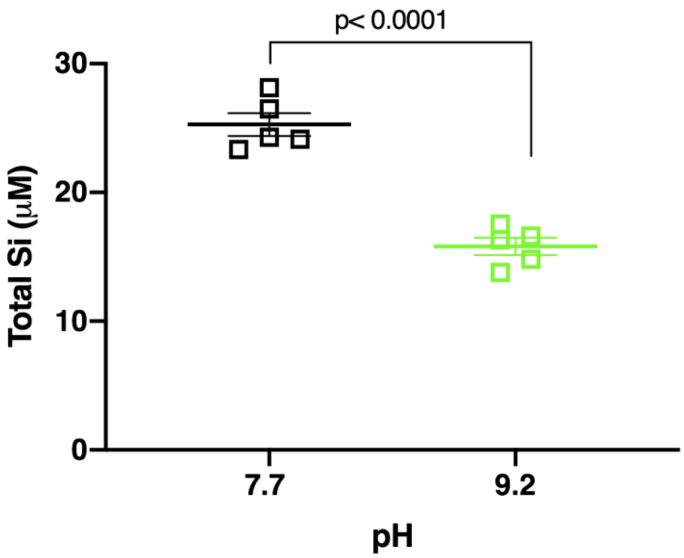

Supplemental Figure 15: Total silica deposited per *F. crotonensis* culture after 48 h PDMPO incubations in pH 7.7 (black squares) and pH 9.2 (green squares) acclimated cultures. Data corresponds to *in vitro F. crotonensis* Si deposition assay. Statistically significant differences are denoted by respective p values generated by unpaired two-tailed t-tests. Standard error of the mean reported by error bars.

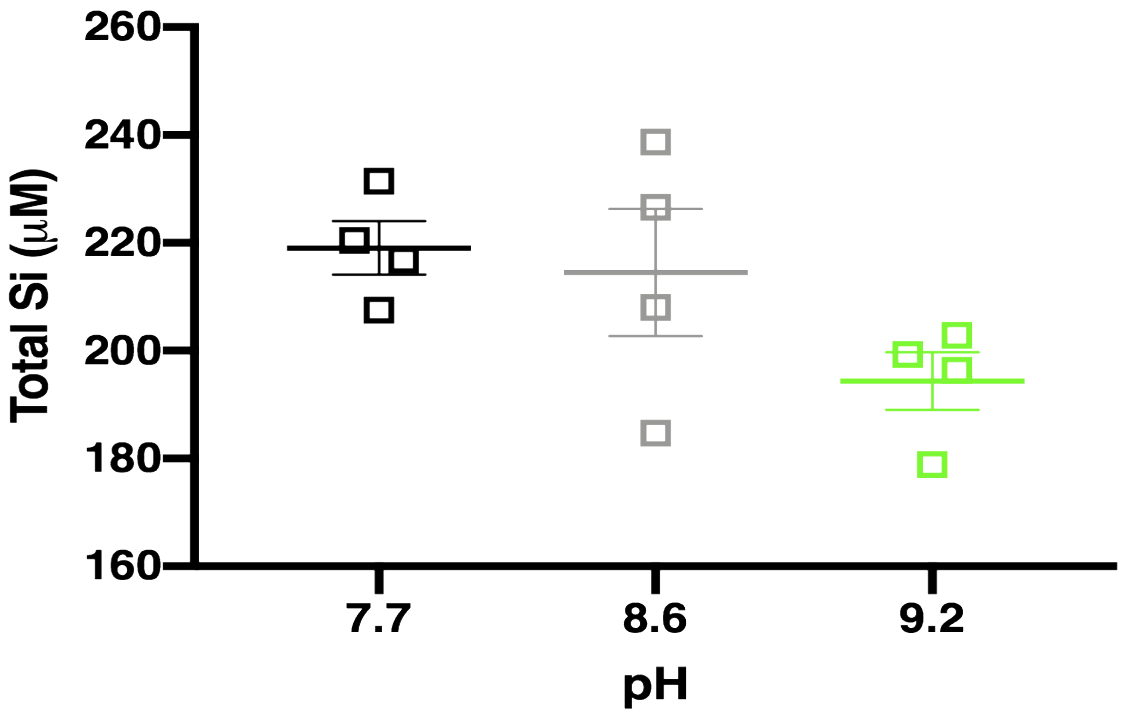

Supplemental Figure 16: Total silica deposited per sample after 48 h PDMPO incubations in pH 7.7 (black squares), pH 8.6 (grey squares) and pH 9.2 (green squares) treatments. Data corresponds to *in situ* Lake Erie Si deposition assay. Statistically significant differences are denoted by respective p values generated by One-way ANOVAs. Standard error of the mean reported by error bars.

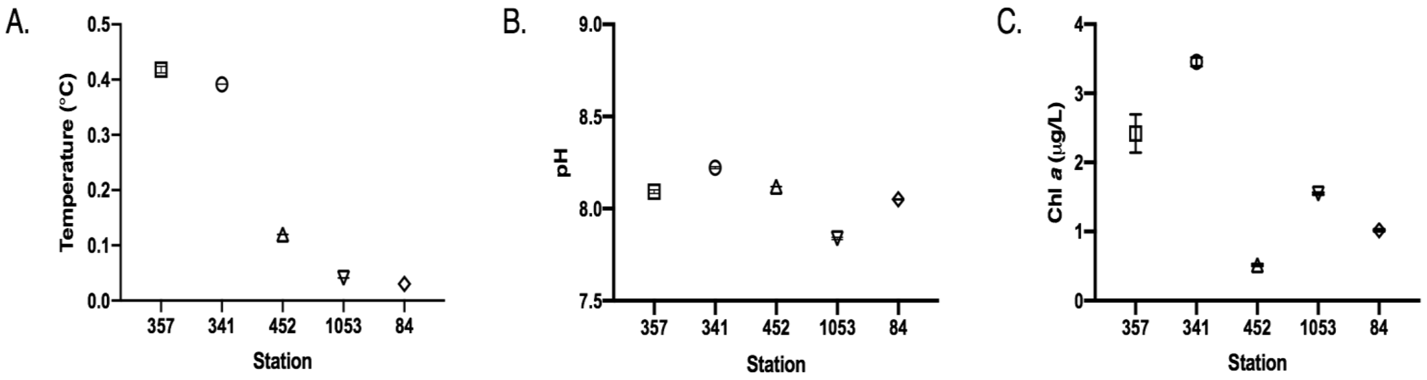

Supplemental Figure 17: Water column profiles collected during a 2-day period (February 17-19) of a 2009 winter cruise in the western-central basins of Lake Erie. Sampling stations correspond to the following: Station 357, 341, 452 (western basin), station 1053, 84 (central basin). **(A)** Mean temperature per station. **(B)** Mean pH per station. **(C)** Mean chlorophyll *a* concentration per station. Standard error of the mean reported by error bars.
